# Mycoviruses in Entomopathogenic Fungi

**DOI:** 10.3390/v17121593

**Published:** 2025-12-08

**Authors:** Matheus da Silva Camargo, Sam Edwards, Maressa O. Henrique, Urja Solanki, Tae Young Shin, Bo Huang, Henrik H. De Fine Licht, Marcio C. Silva-Filho, Augusto Schrank, Robert H. A. Coutts, Ioly Kotta-Loizou

**Affiliations:** 1 Programa de Pós-Graduação em Biologia Celular e Molecular, Centro de Biotecnologia, Universidade Federal do Rio Grande do Sul, Porto Alegre 91501-970, RS, Brazil; silva.camargo@ufrgs.br (M.d.S.C.); aschrank@ufrgs.br (A.S.); 2 Department of Life Sciences, Imperial College London, London SW7 2AZ, UK; 3 Laboratory of Virology, Wageningen University & Research, 6708 PB Wageningen, The Netherlands; sam.edwards@wur.nl; 4 Departamento de Genética, Escola Superior de Agricultura Luiz de Queiros, Universidade de São Paulo, Piracicaba 13418, SP, Brazil; maressahenrique@usp.br (M.O.H.); mdcsilva@usp.br (M.C.S.-F.); 5 Department of Clinical, Pharmaceutical & Biological Science, School of Health, Medicine and Life Sciences, University of Hertfordshire, Hatfield AL10 2AB, UK; u.solanki@herts.ac.uk (U.S.); r.coutts@herts.ac.uk (R.H.A.C.); 6 Department of Agricultural Biology, College of Agricultural and Life Sciences, Jeonbuk National University, Jeonju 54896, Republic of Korea; tyshin@jbnu.ac.kr; 7 Anhui Provincial Key Laboratory of Biological Control, Anhui Agricultural University, Hefei 230036, China; bhuang@ahau.edu.cn; 8 Section for Organismal Biology, Department of Plant and Environmental Sciences, University of Copenhagen, 1871 Frederiksberg, Denmark; hhdefinelicht@plen.ku.dk

**Keywords:** entomopathogenic fungi, mycoviruses, *Beauveria*, *Cordyceps*, *Entomophthora*, *Metarhizium*, *Trichoderma*, *Partitiviridae*, *Chrysoviridae*, *Alphatotiviridae*, biological control

## Abstract

Mycoviruses are widely distributed among different groups of filamentous fungi. An awareness of infections caused by mycoviruses was highlighted in the 1980s and 1990s, when the impact of these agents on phenotypes of agriculturally and medically important fungi was reported. However, for entomopathogenic fungi, mycovirus research has only expanded significantly in the last 15 years. Due to the agricultural importance of these fungi, reflected in their use at the forefront of biological control strategies, recent studies have extensively described novel viruses and their effects on their hosts in terms of altered morphological, phenotypical, and ecological characteristics. To summarise the historical progress of mycovirology and recent discoveries, here we describe the state of the art in the study of mycoviruses associated with entomopathogenic fungi. We have limited the review to the occurrence of mycoviruses in fungi of the genera *Beauveria*, *Cordyceps*, *Entomophthora*, *Metarhizium*, and *Trichoderma* and have compiled an inventory of the viruses reported to infect these entomopathogenic genera, as well as a comprehensive review of the biological effects described with respect to infection by mycoviruses in fungi that are relevant to the biological control of insects. Finally, we have outlined possible research scenarios in the light of recent discoveries in the field of mycovirology, such as the use of mycoviruses as virulence modulating factors: the main character sought in biological pest control.

## 1. Introduction

**Figure 1:**
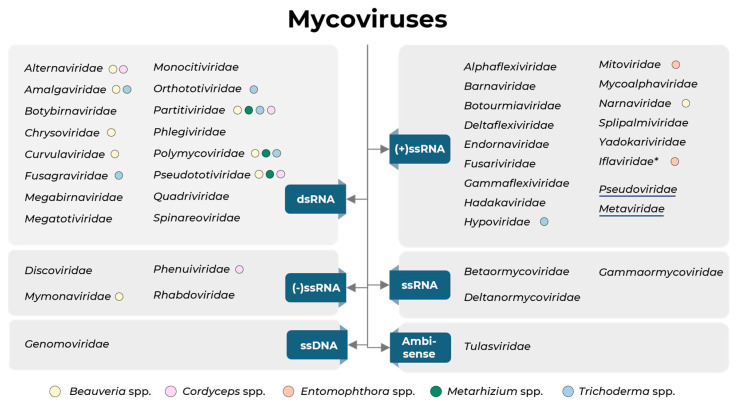
Current organisation of mycoviruses taxonomy based on their genomes. Most families are characterised by dsRNA and (+)ssRNA genomes, including the underlined reverse transcribing families. Four (–)ssRNA, one circular ssDNA, and one ambisense coding families are also recognised. The ssRNA group includes recently established mycoviruses (ourmycoviruses) whose genome polarity could not be defined according to standard criteria. Yellow, lilac, salmon, green, and light blue circles indicate the presence of specific viral families in *Beauveria, Cordyceps Entomophthora*, *Metarhizium*, and *Trichoderma*, respectively. Taxonomic data retrieved from ICTV-Virus Metadata Resource tables (ICTV; ictv.global/vmr; accessed in July 2025). * The family *Iflaviridae*, although not currently classified as a mycovirus family by the ICTV, was included due to its occurrence in *Entomophthora*.

Mycoviruses were first described more than 60 years ago in the edible basidiomycete *Agaricus bisporus* [1], marking the beginning of a new field in fungal biology. In the following decades, particularly during the ‘golden era’ of mycovirology, research efforts intensified toward the discovery and characterisation of viral elements infecting phytopathogenic fungi. This period established the foundation for understanding virus–fungus interactions, their genomic diversity, and their potential influence on fungal physiology. One of the most emblematic cases was that of Cryphonectria hypovirus 1 (CHV1) infecting the chestnut blight fungus *Cryphonectria parasitica*. The introduction of CHV1 into wild fungal populations successfully induced a hypovirulent phenotype, preventing the development of a devastating chestnut blight epidemic across the continent of Europe’s forests, representing one of the earliest and most successful examples of biological control mediated by a mycovirus [2]. In the last decade, along with advances in sequencing techniques and the expansion of the mycovirus field, several new and unclassified viruses were discovered and already established families were expanded, with novel members possessing different characteristics. Viral taxonomy has advanced to such an extent that several reorganisations were made in taxonomic groups to better accommodate the new taxa. According to the International Committee on Taxonomy of Viruses (ICTV; ictv.global/vmr; accessed in July 2025) and a recent update of taxonomy changes from the ICTV Fungal and Protist Viruses Subcommittee [3], there are currently 41 families accommodating viruses infecting fungi (Figure 1).

Currently, the most widely described mycoviruses are those with positive-sense single-stranded RNA ((+)ssRNA) genomes, which comprise 16 families (including the reverse-transcribing families *Pseudoviridae* and *Metaviridae*). Equally abundant are the double-stranded RNA (dsRNA) viruses, which are also distributed across 16 families (Figure 1). Mycoviruses with negative-sense single-stranded RNA ((–)ssRNA) genomes are organised into four families. Ormycoviruses, a group of ssRNA viruses, were also recognised in the year of 2025 by the ICTV Fungal and Protist Viruses Subcommittee. To date, only one single-stranded DNA (ssDNA) family, *Genomoviridae*, has been reported to infect fungi. Finally, the *Tulasviridae* family possesses both positive- and negative-sense strands for RNA translation and is therefore classified as ambisense (Figure 1).

Recently, changes were made to the order *Ghabrivirales*, which includes mycoviruses with dsRNA genomes [4]. The order, which previously included the families *Chrysoviridae*, *Megabirnaviridae*, *Quadriviridae*, and *Totiviridae*, now has a new taxonomic layer at suborder level, subdivided into *Alpha*-, *Beta*-, and *Gammatotivirineae*. To accommodate the fungal viruses previously classified in the *Totivirus* and *Victorivirus* genera, the *Orthototiviridae* and *Pseudototiviridae* families were created in the *Alphatotivirineae* suborder. A new family, *Monocitoviridae*, was created to accommodate the virus Ustilago maydis virus H1, previously belonging to the genus *Totivirus*. Previously proposed families have now been recognised, each including a single genus: family *Botybirnaviridae* and genus *Botybirnavirus*; family *Fusagraviridae* and genus *Fusagravirus*; family *Megatotiviridae* and genus *Megatotivirus*; and, finally, family *Phlegiviridae* and genus *Phlegivirus* [4,5].

In addition to the description of new virus taxonomic groups, genomes, and their molecular characteristics, aspects associated with mycovirus infection, such as phenotypic changes, are of scientific, agricultural, industrial, and medical interest. Effects on virulence, for example, play important roles in host fitness and are, therefore, widely studied. Among the earliest and most successful biotechnological applications of mycoviruses are the dsRNA viruses in *Saccharomyces cerevisiae* that confer the ‘killer yeast’ phenotype. These viruses enable their fungal hosts to secrete toxins that kill competing yeast strains, a trait that has been effectively utilised in industrial fermentation processes, including wine production, beer brewing, and bread making, to prevent contamination by wild yeast strains [6]. Hypovirulence has been mainly observed and used to human benefit in plant-pathogenic fungi [7,8,9]. As mentioned above, *C. parasitica*, the ascomycete responsible for chestnut blight, has been successfully controlled through mycovirus-based treatment, which is considered a model in forest disease management [10]. Conversely, mycovirus infections in *Aspergillus* are widely investigated as molecular tools to alter the virulence of species responsible for aspergillosis, with recent reports indicating that they may either reduce or increase pathogenicity in mammalian hosts [11,12].

The modulation of fungal virulence and fitness observed in medically and agriculturally relevant genera such as *Aspergillus* and *Cryphonectria* [13,14] has increased interest in other fungal groups of agricultural importance. Among these, entomopathogenic fungi (EPF) have gained attention for their potential to exhibit similar virus-induced phenotypic alterations. Considering the recognised role of EPF in sustainable pest management, particular interest lies in the ability of mycoviruses to induce hypervirulent phenotypes that may enhance biological control effectiveness. In this context, exploring mycoviruses as modulators of EPF virulence aligns with the growing global demand for sustainable pest control strategies. Over the past three decades, these fungi have attracted attention as an alternative to reduce the environmental impact of traditional chemical pest control strategies [15]. Emerging environmental and health management strategies, such as the One-Health approach, emphasise the urgent need for pest control strategies that minimise the environmental impact while ensuring effective and robust crop protection [16]. Notably, integrated pest management utilising biological control agents has demonstrated promising outcomes, achieving damage reductions of over 73% across a variety of crops and targeting 43 different invertebrate pests [17]. Amongst several organisms commercially employed as biological control agents, EPF are at the forefront of research interest due to their effectiveness [18,19,20,21]. Mycoinsecticides, biological control products based on EPF, represent 60% of the total of 82 products registered and marketed in Brazil as biological controllers. Of these 82, *Metarhizium* and *Beauveria* lead the list, with 24 and 13 products, respectively [21].

The first investigations of mycoviruses within EPF were carried out in the 1990s and revealed dsRNA elements and viral particles related to mycovirus infections. Given the agricultural importance of EPF, these studies mainly sought to understand how mycoviruses impacted aspects such as sporulation and virulence in arthropods. Complete genome sequences of these mycoviruses only became available in the 2010s, coinciding with a significant increase in the number of new mycoviruses for different genera of EPF. In view of this, this review aims to present the current genomic and functional description of mycoviruses and their relationship with their fungal hosts. The review is focused on EPF genera that are widely used as biological control agents in integrated pest management systems, including *Beauveria*, *Cordyceps*, *Metarhizium*, and *Trichoderma*, as well as the genus *Entomophthora*, which stands out for its obligate association with arthropod hosts and its ecological importance in natural pest regulation.

## 2. Incidence of Mycoviruses in Entomopathogenic Fungi

**Figure 2:**
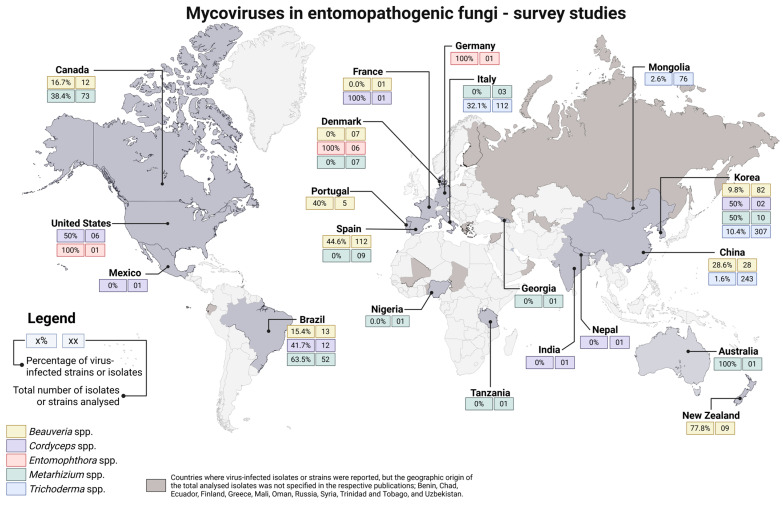
Global map illustrating mycovirus infection rates and distribution in entomopathogenic fungi isolates/strains worldwide. For countries where rate calculation was possible, text boxes indicate the quantitative data used, presented in the following format: [percentage of infected isolates/strains] and [total number of isolates/strains analysed]. Countries shaded in grey represent locations where mycovirus presence in entomopathogenic fungi isolates/strains has been reported in the literature, but the origin of total number of isolates tested in those studies was not specified. Created in BioRender. Camargo, M. (2025) https://BioRender.com/tlpj9ma (accessed on 2 December 2025).

Initial studies on mycoviruses infecting EPF focused on screening for dsRNA elements and virus-like particles (VLPs) [22,23]. Prior to sequencing, viral families were characterised and taxonomically inferred by electrophoretic RNA patterns, transmission electron microscopy (TEM), and protein purification followed by SDS-polyacrylamide gel electrophoresis (PAGE) [24]. A comprehensive survey of studies aimed at analysing the incidence of mycoviruses in entomopathogenic fungal populations across different countries is summarised in Figure 2 and detailed at species level in Table S1.

The screening strategy of strains and isolates infected with mycoviruses, despite providing a general overview of the distribution of viral RNA elements in a group of samples, is limited and has a strong bias towards dsRNA virus infections, specifically. The methodologies employed, based on the nature of dsRNA resistant to S1 nuclease and DNase I treatments, cannot indicate the presence of ssRNA viruses, whether in a positive or negative sense, since the occurrence of ds versions of these viruses are scarce. Therefore, the incidence of viruses in isolates and strains may be underestimated, given the possibility that virus-free strains might carry ssRNA viruses exclusively.

While these pioneer studies were conducted in the late 1990s, the field of mycovirology concerning EPF advanced dramatically over the last 15 years. Notably, the first completely sequenced viral genomes for the entomopathogenic genera *Beauveria*, *Cordyceps*, *Trichoderma, Entomophthora*, and *Metarhizium* were reported in the years 2012 [25], 2017 [26], 2017 [27], 2019 [28], and 2020 [29], respectively.

In this section we will discuss the distribution of putative viral dsRNA segments that have already been identified in different species of five genera of EPF in different parts of the world.

### 2.1. Cordycipitaceae: Beauveria and Cordyceps

The genus *Beauveria* belongs to the order Hypocreales and the family *Cordycipitaceae* [30]. This genus is considered a cosmopolitan genus and a facultative parasite, capable of completing its reproductive cycle either as a pathogen or in free form, as a saprophyte or endophyte [31]. The broad spectrum of hosts highlights the potential of *Beauveria bassiana* as a control agent for various target pests [32], infecting a wide range of arthropods, some of which have agricultural importance [33], including the sweet potato whitefly (*Bemisia tabaci*), the banana weevil (*Cosmopolites sordidus*), the corn leafhopper (*Dalbulus maidis*), and the two-spotted spider mite (*Tetranychus urticae*) [21,33]. *B. bassiana* has 707 insect host species, comprising 521 genera and 149 families from 15 orders. Within crop pests, *B. bassiana* is usually reported as infecting insects of the order Lepidoptera and Coleoptera [33].

In a foundational study, Melzer and Bidochka (1998) investigated EPF from Canadian soils, including 12 isolates of *B. bassiana*. Of these, dsRNA elements were detected in two isolates (16.7%), using cellulose chromatography followed by agarose gel electrophoresis [34]. Dalzoto et al. (2006) examined 13 *B. bassiana* strains by RNA patterns in electrophoresis and reported that two of the thirteen strains (15.4%) yielded extra dsRNA bands (~4.0–0.7 kbp) [35]. TEM of one infected strain revealed 28–30 nm isometric virus-like particles, consistent with a *Totiviridae*-like virus. In this early study, no sequence data were reported, so the viral family assignment was based on particle size and genome structure.

Subsequently, Herrero et al. (2012) performed a larger survey of Iberian *Beauveria* [25]. They analysed 73 *B. bassiana* isolates (from Spain and Portugal, obtained from insects, plants, and soil) by agarose electrophoresis of extracted dsRNA. Several dsRNA elements were found in 54.8% of isolates (40 of 73 strains). The detected dsRNA segments ranged in size from 0.7 to 6 kbp and each infected isolate carried between one and five dsRNA bands. Mixed infections (multiple dsRNA viruses per strain) were common. Notably, no clear association was seen between particular dsRNA profiles and the geographical origin or ecological source of the isolates, likely reflecting the wide dispersal of *B. bassiana* spores [25]. One of the most abundant dsRNA types was fully sequenced and shown to encode a ~5.2 kbp genome of a novel *Totiviridae* member, provisionally named Beauveria bassiana RNA virus 1 (BbRV1) [25].

In a separate investigation, Yie et al. (2014) screened nine *Beauveria* isolates collected in New Zealand and detected dsRNA elements in seven of them, corresponding to a remarkably high infection rate of 77.8% [36]. This analysis, based on dsRNA extraction and gel electrophoresis, revealed the presence of dsRNA viral elements in a non-*B. bassiana* isolate, specifically *Beauveria caledonica*, highlighting that mycovirus infections may extend beyond the commonly studied *B. bassiana* [36].

Later surveys expanded the scope to global isolate collections and new detection methods. Kotta-Loizou and Coutts (2017) examined a “well-characterised panel” of 75 *B. bassiana* isolates from worldwide sources (including Brazil, Ecuador, Greece, Portugal, Russia, Spain, Syria, and Uzbekistan) [37]. Using dsRNA extraction with DNase/RNase treatments and agarose gels, they found that 16 of the 75 strains (21.3%) harboured dsRNA elements. TEM analyses revealed isometric particles ~50 nm in diameter (typical of totiviruses and partitiviruses). Sequencing of the dsRNAs identified viruses related to three known families: *Partitiviridae* (encapsidated multi-segmented dsRNA viruses), *Totiviridae* (encapsidated non-segmented dsRNA viruses), and *Narnaviridae* (capsidless ssRNA non-segmented viruses). In fact, this study reported several novel viruses: two partitiviruses, Beauveria bassiana partitivirus (BbPV) 1 and 2; one totivirus, Beauveria bassiana victorivirus 1 (BbVV1); and an unusually small narnavirus-like element. It also discovered the first Beauveria bassiana polymycovirus (BbPmV1), an unencapsidated multi-segmented dsRNA virus, belonging to the family *Polymycoviridae*, formerly “Tetramycoviridae”. Interestingly, 11 of the 16 dsRNA-positive isolates came from arthropod hosts; no particular host or region was consistently associated with infection [37].

Filippou et al. (2018) conducted a population-level screen of European EPF (primarily Spanish and some Danish isolates) [38]. The study focused on testing a large panel of 151 isolates, covering several genera, including *Beauveria*, and used dsRNA gel profiles to identify infected isolates, followed by reverse transcription polymerase chain reaction (RT-PCR) and sequencing to classify the viruses. This study found that 12 of the Spanish *B. bassiana* isolates carried dsRNA viruses. All viruses detected belonged to three known taxa: the previously described BbVV1, BbPV2, and BbPmV1. Some isolates harboured up to three of these viruses simultaneously. No novel families were reported in this survey [38].

More recent work has both confirmed and extended these patterns. Ning et al. (2022) showed experimentally that dsRNA viruses are ubiquitous across *Beauveria* spp. and readily transmitted between species [39]. Although this was not a survey of field isolates per se, it implies that other *Beauveria* species (e.g., *B. amorpha*, *B. aranearum*) also commonly carry dsRNA viruses, and that interspecific spread is efficient. Finally, Shi et al. (2024) surveyed 28 *B. bassiana* strains from the Guniujiang Nature Preserve in Anhui, China. Electrophoretic profiling revealed dsRNA in 8 of 28 isolates (28.6%) [40]. By combining dsRNA gel profile analysis, metatranscriptomic sequencing and RT-PCR, they identified six distinct virus species infecting these strains, including two novel viruses: Beauveria bassiana victorivirus 2 (BbVV2, a new totivirus) and Beauveria bassiana bipartite mycovirus 2 (BbBMV2, a new partitivirus). Four additional viruses matched previously described types, including Beauveria bassiana polymycovirus 4 (BbPmV4), etc. [40]. EPF collected in South Korea were also investigated by Hwang et al. (2023) [41]. The incidence of putative mycoviruses was evaluated in 94 isolates, including 82 isolates of *Beauveria*. dsRNA was detected in 14 out of 94 strains overall (14.9%), and in 8 out of the 82 *Beauveria* isolates (9.8%), specifically [41]. Detection was based on dsRNA profiling via cellulose chromatography and electrophoresis. Although no TEM or sequencing was performed, the authors inferred likely viral families based on the number and size of dsRNA segments: partitivirus-like profiles (two segments of ~1.5–2.2 kbp) were observed in some strains, while single ~5–7 kbp segments suggested the presence of totiviruses. Other isolates, such as *B. bassiana* 261, harboured up to 12 dsRNA segments, suggestive of multipartite viruses like polymycoviruses or chrysoviruses [41].

Within the *Cordyciptaceae* family, the genus *Cordyceps* now includes the former genus *Isaria* and species previously described in the *Paecilomyces* genus, such as *Cordyceps farinosa*, *C. fumosorosea*, and *C. javanica* [42,43]. *Cordyceps* comprises more than 700 species usually isolated from tropical forests and humid climates [44]. Many species are recognised due to their medical and biotechnological relevance [45]; however, the genus is recognised as a typical feature of the entomopathogenic lifestyle. *C. farinosa* and fungi within the *C. fumosorosea* complex are the main species in the genus recognised as biological controllers and exhibit a cosmopolitan distribution. These fungi have been identified across multiple continents, including North and South America, Europe, Africa, Asia, and Australia. The host range of *Cordyceps* spp. is notably broad, encompassing arthropods from multiple orders including Acari, Coleoptera, Diptera, Hemiptera, Hymenoptera, Lepidoptera, and Thysanoptera [42]. *C. fumosorosea*, for example, has demonstrated high efficacy against whiteflies (*Bemisia tabaci*), aphids (*Aphis* spp.), and thrips (*Frankliniella occidentalis*), which are major pests in vegetable and ornamental crops [42,46,47,48].

Studies on the incidence of viral dsRNA or viral particles in fungi belonging to the *Cordyceps*-*Isaria*-*Paecilomyces* cluster are relatively scarce. The first studies were performed simultaneously with the *Metarhizium* surveys detailed earlier. A 52.6% (10/19) incidence of dsRNAs was observed in a group of *Cordyceps* spp. (formerly *Paecilomyces* spp.) culture collection strains from Brazil, France, and the USA, including *P*. *fumosoroseus*, *P*. *amoenoroseus*, and *P*. *farinosus* [49]. Two other studies showed an incidence of viral dsRNA in 25% (3/12) and 50% (1/2) of the *C*. *fumosorosea* strains from different parts of the world [41,50]. Recently, an unpublished study in China revealed that out of 36 randomly selected strains of *C. chanhua* from the RCEF library, 33.3% contained dsRNA segments after extraction using cellulose CF-11. Notably, there is no trend in the occurrence of infected strains among the different countries reported.

### 2.2. Clavicipitaceae: Metarhizium

The genus *Metarhizium* belongs to the order Hypocreales and the family *Clavicipitaceae* [51,52]. Among the different species in the genus, *Metarhizium anisopliae* stands out as one of the most well studied and widely applied [53,54]. *M. anisopliae* has been isolated from >200 species of arthropods and comprises 33.9% of microbial pesticides based on EPF [55]. *M. anisopliae* strains are considered to be host generalists and therefore are effective against a wide range of arthropod pests belonging to the orders Lepidoptera, Hemiptera, Coleoptera, and Diptera [55]. In addition to generalist species, the genus *Metarhizium* harbours insect host order-specialist species, such as *M. acridum* and *M. album*, which are, respectively, infecting arthropods of the orders Orthoptera and Hemiptera [56]. In Brazil, an important producer of global crops, *M. anisopliae* is used on a large scale to control a complex of leafhoppers, including *Mahanarva fimbriolata* and *Mahanarva posticata* in sugar cane crops, and *Mahanarva fimbriolata*, *Deois flavopicta*, and *Notozulia entreriana* in pastures [21].

The incidence of mycoviruses in *Metarhizium* spp. was first reported by Leal et al. (1994) in *M. anisopliae* [23]. Among 41 strains obtained from different parts of the world, dsRNA segments were detected in only 2 (*ca*. 4.8%) Brazilian *M. anisopliae* strains by electrophoretic patterns of RNA extracts. The two strains, V215 and V291, harboured 13 and 9 dsRNA segments ranging from 0.9 to 3.5 kbp in size, respectively, which were associated with isometric VLPs *ca*. 33 nm in diameter, as observed by TEM. It was suggested by the authors that both strains were probably infected with members of the family *Partitiviridae*, as inferred from the VLP profile; however, due to the atypical pattern of bands, no conclusions were made regarding taxonomic placement [23]. In 1996, Bogo et al. [22] also observed particles *ca*. 35 nm in diameter in a Brazilian *M. anisopliae* strain harbouring dsRNA bands varying from 0.9 to 3.1 kbp in size, with a similar electrophoretic banding profile pattern. In this investigation, three out of seven strains (42.9%) contained dsRNA. Additional experiments with Canadian strains reported higher incidences of 38.3% of dsRNA segments and highlighted the presence of a doublet of segments of 2.0 and 1.8 kbp, further indicating the presence of partitiviruses in North American *M. anisopliae* [34,57].

In complementary studies, Bogo et al. (1996) reported the first description of a virus from the then *Totiviridae* family [22]. The presence of a single dsRNA band *ca*. 4 kbp in size in *M. anisopliae* strain M5 was also noted in this early investigation. However, the virus particle profile and characterisation of viral proteins were not described in detail until 2002, when Giménez-Pecci et al. [24] reported the presence of icosahedral particles 35 and 43 nm in diameter which were associated with an abundant protein *ca*. 80 kDa in size: these features were consistent with this virus being a member of the *Totiviridae* family [24].

Perinotto et al., in 2014, reported a high incidence of dsRNA segments in Brazilian strains of *M. anisopliae* [58]. The study revealed the presence of viral infections in four out of five (80%) strains [58]. Similarly, Santos et al. (2017) observed dsRNA segments in 22 (62.8%) out of 35 *M. anisopliae* Brazilian strains [59]. In contrast, in 2018, Filippou et al. failed to detect any dsRNA bands in a large collection of European strains, including *M. anisopliae* [38].

Other species of *Metarhizium*, including *M. flavoviride*, *M. robertsii*, *M. brunneum*, *M. guizhouense*, and *M. majus*, were also investigated by Filippou et al. However, no dsRNA bands were observed in any of these strains [38]. In comparison, a separate study conducted in Korea detected dsRNA bands in three species: *M. pemphigi*, *M. pinghaense*, and *M. rileyi* [41].

Globally, a distinct pattern in the distribution of infected and virus-free *Metarhizium* spp. strains is evident. A higher incidence of dsRNA segments is consistently observed in American strains as compared to European strains. Among studies that specify the locality of isolates, Brazilian strains exhibit an incidence of *ca*. 61.7% (*n* = 47), followed by 38.3% (*n* = 73) observed in Canadian strains. Conversely, surveys of European strains, which included a total of 20 isolates from various *Metarhizium* species, have reported no detection of dsRNAs.

### 2.3. Hypocreaceae: Trichoderma

The genus *Trichoderma* stands out as a versatile biocontrol agent, with applications in formulations as both a mycopesticide and an insecticide [60]. Studies have highlighted its high efficacy against various arthropods, with mortality rates reaching up to 100% in some cases, such as against the red flour beetle (*Tribolium castaneum*) and the red spider mite (*Tetranychus urticae*) [61]. Additionally, *Trichoderma* has been shown to induce sublethal effects, which are also crucial for mitigating crop damage [60].

Belonging to the order Hypocreales and family *Hypocreaceae*, *Trichoderma* has two very distinct parasitism styles that are of significant biotechnological interest [60]. Several studies demonstrate the entomopathogenic potential of *Trichoderma* against insects of considerable agricultural significance, including species from the orders Hemiptera, Coleoptera, and Lepidoptera [60,61]. In relation to the parasitism of other fungi (known as mycoparasitism), *Trichoderma* spp. is widely used due to its ability to rapidly colonise the rhizosphere, which helps to suppress invading pathogens when biocontrol fungi are applied to seeds or roots [62,63,64]. In direct antibiosis, the secondary metabolites or enzymes secreted by *Trichoderma* inhibit the growth or germination of the pathogen [65]. Commercially, *Trichoderma* is widely used as a biopesticide in pest control strategies and consists of 50–60% of commercial biofungicides in the global market [66]. In Brazil, *Trichoderma* represents the third most used mycopesticide registered [21].

Most strains studied for the presence of dsRNA viruses in *Trichoderma* are Asian. In 623 *Trichoderma* spp. strains derived from surveys in different countries, including China, Mongolia, and Korea, only 35 (5.6%) were found to contain dsRNAs. A subsequent study conducted in Italy, however, revealed a higher incidence of dsRNAs in certain species—49.7% (19/39) in *T. harzianum*, 24.1% (7/29) in *T. gamsii*, and 66.7% (6/9) in *T. hamatum*—despite the low sample size. Other species analysed had a low incidence (<5%) or the sample number was less than five.

### 2.4. Entomophthoraceae: Entomophthora

The genus *Entomophthora* belongs to the order Entomophthorales, family *Entomophthoraceae* in the phylum Zoopagomycota [67,68]. Together with Mucormycota, Blastocladiomycota, and Chytridiomyctota, Zoopagomycota belong to the early-diverging fungal lineages outside Dikarya (Ascomycota and Basidiomycota). As such, species in the genus *Entomophthora* are distantly related to the previously mentioned ascomycete EPFs [31], which is also exemplified in their biology.

Fungi in the genus *Entomophthora* are fungal pathogens on a variety of insects: in particular, Diptera and Hemiptera, but also Plecoptera, Thysanoptera, and Raphidioptera [69]. These fungi are obligatory insect pathogens that only grow actively inside insects and often exclusively rely on host-to-host transmission or in temperate regions overwinters as dormant resting spores. Nearly all *Entomophthora* fungi follow a common life cycle consisting of host infection and proliferation in the living host. As the nutritional resources inside the host dwindle, the fungus eventually kills the host, grows out from the inside, and releases conidia that needs to reach a new host. Certain fungi in this genus, such as, for example, *E. muscae*, are famous for altering the behaviour of their host to position the host ideally for spore dispersal [70]. In contrast to ascomycete EPF’s, fungi in the genus *Entomophthora* are true host specialists, often only infecting a single or very few host species [71,72,73]. Because of this host specificity, these fungi have often been suggested as being ideal for biological control of insect pests in agriculture, such as the spotted-wing *Drosophila* [74], but unsuccessful efforts to mass-produce conidia have so far hindered their use in classical biological control. Significantly more success has been achieved using conservational biological control methods, exemplified with the related *Entomophaga maimaiga* against the gypsy moth (*Lymantria dispar*) in the eastern USA [75].

Although the study of viruses in the *Entomophthoraceae* is still in its infancy, transcriptome-mining analyses are revealing that the *Zoopamycota* phylum has one of the highest prevalences (up to *ca*. 55%) and diversity of RNA viruses across the Fungal Kingdom [76,77]. These viruses are related to seven known families: *Benyviridae* (encapsidated non-segmented (+)ssRNA viruses), *Iflaviridae* (encapsidated non-segmented (+)ssRNA viruses), *Mitoviridae* (capsidless (+)ssRNA non-segmented viruses), *Narnaviridae*, *Virgaviridae* (encapsidated segmented or non-segmented (genus dependent) (+)ssRNA viruses), *Rhabdoviridae* (encapsidated non-segmented (−)ssRNA viruses), and *Partitiviridae*. Of these, only the *Iflaviridae* and *Mitoviridae* have been studied more deeply, showing ubiquitous associations with *Entomophthora muscae* across all known isolates, bar one for *Iflaviridae* [28,78,79]. As most molecular studies on *Entomophthora* have been transcriptomic, only RNA viruses have been identified to date, leaving opportunities open for identifying DNA viruses in future studies.

## 3. The EPF Virome

**Figure 3:**
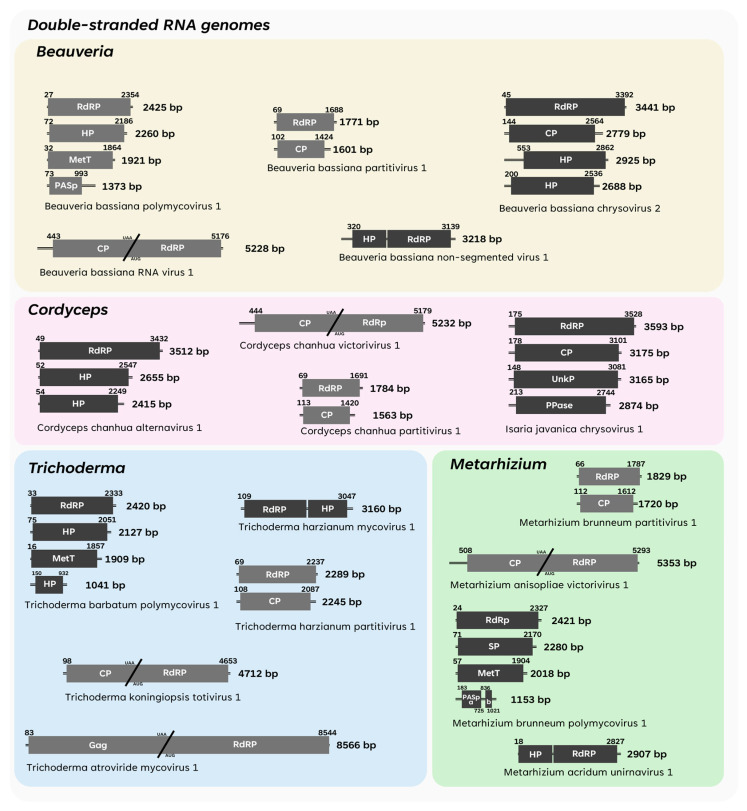
Genome architecture of representative dsRNA mycoviruses described in *Beauveria*, *Cordyceps*, *Entomophthora*, *Metarhizium*, and *Trichoderma*. Only one representative virus is shown per viral taxon. Viral genomes are grouped by fungal host genus, each highlighted within coloured boxes: *Beauveria* (yellow), *Cordyceps* (lilac), *Entomophthora* (salmon); *Metarhizium* (green), and *Trichoderma* (blue). RdRP: RNA-dependent RNA polymerase; CP: coat protein/capsid; HP: hypothetical protein; Hel: helicase; MetT: Methyltransferase; PASrp: proline-alanine-serine-rich protein; UnkP: protein of an unknown function; PPase: putative protease; and Gag: putative structural/gag protein.

**Figure 4:**
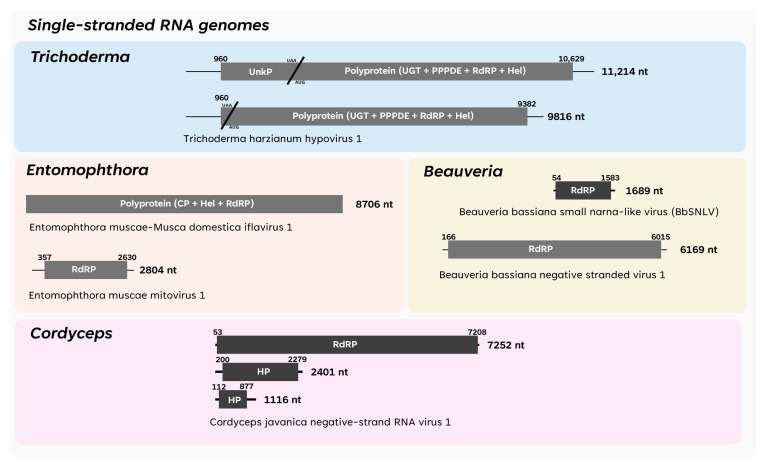
Genome architecture of representative ssRNA mycoviruses described in *Beauveria*, *Cordyceps*, *Entomophthora*, and *Trichoderma*. Only one representative virus is shown per viral taxon. Viral genomes are grouped by fungal host genus, each highlighted within coloured boxes: *Beauveria* (yellow), *Cordyceps* (lilac), *Entomophthora* (salmon), and *Trichoderma* (blue). RdRP: RNA-dependent RNA polymerase; CP: coat protein/capsid; HP: hypothetical protein; Hel: helicase; UGT: UDP-glucose/sterol glucosyltransferase domain; and PPPDE: putative domain of permuted papain fold peptidases.

The following section presents the history of mycoviruses identified in EPF. The first characterised viruses for each fungal genus are mentioned, and the complete list of fully described viruses is shown in Tables S2–S6. Additionally, two schematic figures are provided to visually summarise all the representative dsRNA (Figure 3) and ssRNA (Figure 4) viral genomes described to date in *Beauveria*, *Cordyceps*, *Entomophthora*, *Metarhizium*, and *Trichoderma*, with each fungal genus being distinguished by a specific colour-coded box.

### 3.1. dsRNA Viruses

#### 3.1.1. Pseudototiviridae and Orthototiviridae

The former family *Totiviridae*, which included the *Totivirus* and *Victorivirus* genera, was reorganised into three new suborders within the *Ghabrivirales* order. In the *Alphatotivirinae* suborder (*Chrymotiviricetes*, *Ghabrivirales*), both the *Orthototiviridae* and *Pseudototiviridae* families were accommodated and now comprise the *Totivirus* and *Victorivirus* genera, respectively. Both genera are characterised by their ability to infect fungi, the possession of isometric virions *ca*. 40 nm in diameter and a monosegmented dsRNA genome, ranging from 4.6 to 6.7 kbp. The genome encodes both an RdRP and a CP, with expression typically occurring via ribosomal frameshifting.

The first member of the genus *Victorivirus* found infecting *B. bassiana* was named Beauveria bassiana victorivirus 1 (BbVV1). The virus was reported as a single dsRNA band of *ca*. 6 kbp with isometric particles of *ca*. 50 nm in diameter. Following complete genome sequencing, the 5327 bp dsRNA was found to have two overlapping open reading frames (ORFs) encoding, respectively, a CP and an RdRP [36]. Recently, *C. chanhua* was found to be coinfected with Cordyceps chanhua alternavirus 1 (CcAV1) and Cordyceps chanhua victorivirus 1 (CcV1). The virus has two ORFs: ORF1 encodes a putative CP of 742 amino acids, and ORF2 encodes a putative RdRP of 836 amino acids. The termination codon of the CP ORF overlaps with the initiation codon of the RdRP ORF at the sequence AUGA [80].

A second genome of the genus *Victorivirus*, 5353 bp in length, was also identified in *M. anisopliae* and named Metarhizium anisopliae victorivirus 1 (MaVV1). The genome comprises two ORFs encoding a CP and an RdRP, respectively. Overlapping both ORFs, the presence of an octanucleotide (AUGAGUAA) as a frameshifting site was observed [81].

In *Trichoderma*, notably, a virus from the *Totivirus* genus has been fully described as Trichoderma koningiopsis totivirus 1 (TkTV1). Despite being in another genus and recently accommodated in another family, the general characteristics of TkTV1 resemble the victoriviruses described in *Beauveria* and *Metarhizium*, such as the presence of two overlapping ORFs responsible for encoding CP and RdRP, a genome *ca*. 5 kbp in size and viral particles *ca*. 40 nm in diameter [82].

#### 3.1.2. Amalgaviridae

*Amalgaviridae* (class *Duplopiviricetes*, order *Durnavirales*) is a family of dsRNA viruses originally known from plants but now also encompassing mycoviruses infecting fungi. Members of this family have non-segmented genomes (*ca*. 3–3.5 kbp) with two not-obligated overlapping ORFs encoding an RNA-dependent RNA polymerase (RdRP) and a smaller protein of an unknown function. The family was recently updated and now includes three distinct genera: *Amalgavirus*, *Unirnavirus*, and *Zybavirus.* The genus *Unirnavirus* includes exclusively non-segmented dsRNA mycoviruses with their occurrence restricted to filamentous fungi. These fungal viruses share a characteristic genome organisation with two ORFs arranged in different reading frames, requiring a ribosomal frameshift for expression of the two proteins. In contrast to many dsRNA viruses, no true virion particles have been observed for unirnaviruses. For instance, Beauveria bassiana non-segmented virus 1 (BbNV1) was the first unirnavirus described from *B. bassiana*: the BbNV1 genome is 3218 bp in length and encodes two proteins, including an HP with no predicted function (315 aa) and an RdRP (585 aa) [83]. A similar virus was identified in *M. acridum* (Metarhizium acridum unirnavirus 1), which is 2907 bp in size and encodes a HP and an RdRP of 317 aa and 609 aa, respectively [84]. In *T. harzianum*, Trichoderma harzianum mycovirus 1 (ThMV1) displays a slightly distinct genomic arrangement. Its genome is 3160 bp in length and contains two major ORFs on the negative strand: ORF-A encodes an RdRP of 582 aa, while ORF-B encodes a HP of 379 aa. Additionally, a third ORF (ORF-C) is located on the positive strand and encodes a hypothetical 94 aa protein, with a poly(A) structure at the 3′ terminus [85].

#### 3.1.3. Fusagraviridae

The family *Fusagraviridae*, accommodated into the new suborder *Alphatotivirinae* (*Chrymotiviricetes*, *Ghabrivirales*—ICTV release 2023), was first proposed in 2016 to include *Fusarium*-related mycoviruses [86] and harbours a unique genus, *Fusagravirus*. This family is characterised by non-segmented dsRNA genomes *ca*. 11 kbp in size, with two ORFs encoding a protein with RdRP domains and a structural protein with potential capsid function (yet some studies have suggested a capsidless nature for fusagraviruses) [87,88]. Similarly to alphatotiviruses, fusagraviruses possess isometric VLPs *ca*. 40 nm in diameter [89] and a ribosomal frameshift system between both ORFs. Moreover, although fusagraviruses are mainly described as infecting filamentous fungi, the host range expands to include plants and oomycetes [86].

A unique fusagravirus, Trichoderma atroviride mycovirus 1 (TaMV1) has been identified in *T. atroviride.* True virions of TaMV1 are encapsidated in icosahedral particles *ca*. 40 nm in diameter and possess a monosegmented dsRNA genome of 9584 bp, encoding both a structural/Gag protein and an RdRP [90].

#### 3.1.4. Partitiviridae

The family *Partitiviridae* (*Duplopiviricetes*, *Durnavirales*) includes five genera, three of which—*Alpha*-, *Beta*-, and *Gammapartitivirus*—are primarily found in fungal hosts [91]. Characteristically, viruses in this family possess small, bisegmented dsRNA genomes ranging in size from 3.0 to 4.8 kbp in total [92]. Each segment is separately packaged into non-enveloped, isometric virions ranging from 25 to 43 nm in diameter [93].

As mentioned above, partitiviruses mark the beginning of the description of putative mycoviruses in EPF. However, the first complete genome belonging to a member of the *Partitiviridae* family found infecting an EPF was only reported in 2017. Beauveria bassiana partitivirus 1 (BbPV1) was identified infecting *B. bassiana* and was shown to possess a bi-segmented dsRNA genome 1771 bp and 1601 bp in length, encoding an RNA-dependent RNA polymerase (RdRP) and a coat protein (CP), respectively. Phylogenetic analyses placed the virus in the genus *Gammapartitivirus* [37].

With the advent of sequencing and next-generation sequencing (NGS) platforms, recent studies have accelerated, defining the incidence and genome sequences of partitiviruses in EPF. Trichoderma harzianum partitivirus 1 (ThPV1), a member of genus *Betapartitivirus*, was described in *T. harzianum*, consisting of two dsRNA segments, 2289 bp and 2245 bp in length, that encode an RdRP and CP, respectively [94]. In *Metarhizium*, the first complete genome of a mycovirus published in the genus was identified infecting *M. brunneum* and described as a bipartite genome of 1829 bp and 1720 bp, encoding RdRP and CP separately [29].

In *Cordyceps*, recent research has expanded the classification of partitiviruses, unveiling novel members in this genus. Cordyceps militaris partitivirus 1 (CmPV1) was discovered in *C. militaris*, identified as a new member of the *Betapartitivirus* genus, with two dsRNA segments encoding RdRp and CP. Notably, dsRNA1 has the highest similarity to that of Bipolaris maydis partitivirus 2 (BmPV2), whereas dsRNA2 shows the highest similarity to human blood-associated partitivirus (HuBPV) [95]. This finding holds significant evolutionary implications, since there is a similarity with mycovirus infecting distant hosts (phytopathogenic/entomopathogenic fungi ↔ humans). Meanwhile, Cordyceps chanhua partitivirus 1 (CchPV1) was documented in *C. chanhua*, classified within the *Gammapartitivirus* genus [96].

Finally, underpinned by numerous previous studies that have extensively described the genomic and taxonomic characteristics of the *Partitiviridae* family, the most recent descriptions of partitiviruses infecting EPF detail the incidence of a *Gammapartitivirus* [97], an *Alphapartitivirus* [98], a *Betapartitivirus* [94], and a proposed “Epsilonpartitivirus” [29] genera, indicating significant genome diversity.

#### 3.1.5. Curvulaviridae

The *Curvulaviridae* family belong to the order *Durnavirales* (class *Duplopiviricetes*), which was first described in a strain of *Curvularia protuberata*. Genomes of the *Curvulaviridae* family are bisegmented and consist of two dsRNA segments: dsRNA1, *ca*. 2.0–2.4 kbp in size, and dsRNA2, ranging in size from 1.7 to 2.0 kbp. The larger segment (dsRNA1) encodes a putative RdRP, while the smaller segment (dsRNA2) encodes one or two proteins of unknown functions. Curvulaviruses were found to be encapsidated in spherical virions measuring 26–29 nm in diameter, and exemplars occur in fungi exclusively (ICTV taxonomic details available at the following: Taxon Details | ICTV). In *B. bassiana*, the dsRNA genome of a virus from the genus *Orthocurvulavirus* was described as containing two segments, comprising one ORF each. The first segment is 6169 bp long and possesses an ORF coding for an RdRP. The second is 1765 bp long and encodes a CP [99].

#### 3.1.6. Chrysoviridae

The family *Chrysoviridae* (*Ghabrivirales*, *Alphatotivirineae*) is characterised by multisegmented dsRNA-based genomes. The virus particles are isometric, small (35–40 nm in diameter), and non-enveloped [100]. Typically, chrysoviruses possess four genomic dsRNA segments, with a total genome size ranging from 8.9 to 16.0 kbp, which are encapsidated individually in separate particles [101]. The host distribution includes ascomycetous and basidiomycetous fungi, plants, and possibly insects [101]. The family is divided into two genera: *Alphachrysovirus*, with 20 species, and *Betachrysovirus*, with 11 species (https://talk.ictvonline.org/taxonomy/, accessed on 7 January 2025).

The first associations of the presence of chrysoviruses in EPF were reported by Herrero et al. (2012) in *B. bassiana* [25], where the presence of groups of bands formed by four segments between 2.4 and 3.6 kbp was detected [25]. The first complete genome sequence, however, was reported as infecting a strain of *Isaria javanica* (*Cordycipitaceae*). Isaria javanica chrysovirus 1 (IjCV1), phylogenetically related to the alphachrysovirus Aspergillus fumigatus chrysovirus (AfuCV), contains four dsRNA segments of 3.7, 3.2, 3.1, and 2.8 kbp, encoding RdRP, CP, and two hypothetical proteins, HP1 and HP2, respectively [26]. Similar chrysoviruses are also described as infecting *B. bassiana*, Beauveria bassiana chrysovirus (BbCV) 1 and 2, which belong to the genus *Alpha*- and *Betachrysovirus*, respectively [102,103].

#### 3.1.7. Alternaviridae

The *Alternaviridae* family belongs to the order *Ghabrivirales* (suborder *Gammatotivirineae*) and is characterised by a genome ranging in size from 8.4 to 10.7 kbp in total, divided into 3–4 dsRNA segments. The largest segment (dsRNA1) contains a single ORF encoding the RdRP. The dsRNA2 encodes a conserved protein of an unknown function, while dsRNA3 encodes a CP. When present, the fourth segment (dsRNA4) contains an ORF for a non-conserved protein with an unknown function, often lacking homology among other alternaviruses. Morphologically, alternaviruses are isometric virions *ca*. 33 nm in diameter.

To date, alternaviruses have been found exclusively in ascomycetes. In *C. chanhua*, Cordyceps chanhua alternavirus 1 (CcAV1) was described as containing three dsRNA segments, the first being 3512 bp long and encoding an RdRP, and the second and third encoding hypothetical proteins (HP) of 2655 and 2415 bp in length [104].

#### 3.1.8. Polymycoviridae

The first member of the family *Polymycoviridae* (unclassified viruses within the realm *Riboviria*) was reported in 2015 and named Aspergillus fumigatus tetramycovirus 1. This family consists of a single genus, *Polymycovirus*, and is characterised by multisegmented dsRNA genomes, ranging from 7.5 kbp to 12.5 kb with four to eight segments. These viruses, in their majority, are non-conventionally encapsidated, and fungi and oomycetes serve as their natural hosts [105]. Typically, polymycoviruses’ genomes include segments that encode a RdRP, a methyltransferase (MetT), and a proline-alanine-serine-rich protein (PASrp). The other segments encode proteins of an unknown function.

In EPF, the first description of a polymycovirus, Beauveria bassiana polymycovirus 1 (BbPmV1) was reported in *B. bassiana* [37]. The observation of genomic dynamics in terms of the number of segments and sequence homology with previously described viruses led to the need to create a new family called *Polymycoviridae*. BbPmV1 consists of four dsRNA segments, which were 2425 bp, 2260 bp, 1921 bp, and 1373 bp in length, respectively. The segments encode an RdRP, a protein of an unknown function containing a cysteine-rich zinc finger-like motif, a MeT, and a PASrp, respectively. For BbPmV1, neither structural proteins were observed nor true virions by TEM [37]

In *Metarhizium*, two polymycoviruses were fully described, Metarhizium brunneum polymycovirus 1 (MbPmV1) and Metarhizium anisopliae polymycovirus 1 (MaPmV1), which both contain four dsRNA segments coding for an RdRP, a MetT, a protein of an unknown function, and a PASrp, respectively [106,107]. A similar pattern is found in Trichoderma barbatum polymycovirus 1 (TbPMV1), which contains four segments: RdRP, hypothetical protein 1 (HP1), MetT, and HP2 [108].

### 3.2. ssRNA Viruses

The occurrence of ssRNA viruses in EPF is still rare. As a consequence of the methods used to assess the occurrence of mycoviruses in fungal strains and isolates, based on the treatment of total RNA with S1 nucleases, the availability and quantity of ssRNA material can be limited for detection and sequencing techniques.

The first and smallest ssRNA virus described so far in EPF is Beauveria bassiana’s small narna-like virus (BbSNLV), which consists of a single (+)ssRNA segment, 1689 nt long, coding an RdRP. BbSNLV is a member of family *Narnaviridae* and order *Wolframvirales*, typically comprising a capsidless single segment of (+)ssRNA, up to 3 kbp in length, coding for an RdRP. Other features of viruses in the *Narnaviridae* family include their broad host range, encompassing fungi, algae, protozoans, and invertebrates, as well as their ability to replicate within organelles such as mitochondria, a characteristic observed exclusively in mitoviruses (family *Mitoviridae*) [109].

Similarly, possession of a single genome segment is a characteristic of Beauveria bassiana negative-strand RNA virus 1 (BbNSV1), which consists of a single negative-sense ssRNA strand of 6169 nt, phylogenetically placed in a clade close to viruses of the *Mymonaviridae* family (order *Mononegavirales*). The genomes of mymonaviruses contain a single *ca*. 10,000 nt long (−)ssRNA, potentially encoding up to six proteins [110,111]. Most of the previously described mymonaviruses encode an RdRP, a nucleocapsid (NC) protein, an HP, and several proteins of an unknown function. Mymonaviruses are enveloped filamentous virions, measuring 25–50 nm in diameter and *ca*. 1000 nm in length [112]. Mymonavirids usually infect filamentous fungi, and they have also been reported in oomycetes, plants, and insects following metagenomic studies [111]. A second (−)ssRNA virus was isolated from *C. javanica* and was named Cordyceps javanica negative-strand RNA virus 1 (CjNRSV1). The virus belongs to the little studied genus *Laulavirus* in family *Phenuiviridae* (order *Hareavirales*). The genome of CjNRSV1 is tri-segmented and consists of RNAs 1, 2, and 3, which are 7252, 2401 and 1117 nt in length, respectively. RNA1 encodes an RdRP; RNA2 encodes a non-structural HP, which resembles the movement protein (MP) found in plant viruses; and RNA3 encodes the NC protein [113]. Although the function of RNA2 is still unclear, some studies have reported this ORF as encoding a non-structural protein resembling the movement protein (MP) (ICTV, release 2023). The host range of laulaviruses remains unclear, as current evidence is limited to metagenomic analysis of plant-associated fungi [114] and pools of tick specimens [115]. Therefore, it is proposed that these viruses may be either specific to fungi or ticks.

In *Trichoderma*, the only ssRNA viruses described thus far belong to the *Hypoviridae* family (order *Durnavirales*). This family comprises capsidless viruses with (+)ssRNA genomes, ranging from *ca*. 7.3 to 18.3 kb in length. Hypoviruses lack true virions; their RNA genomes are encapsulated within vesicles derived from the host’s Golgi apparatus. This unique feature distinguishes them from other mycoviruses. The host range of hypoviruses is limited to fungi.

Trichoderma harzianum hypovirus 1 (ThHV1) is characterised by a (+)ssRNA strand of 11,214 nt, containing two overlapping ORFs. ThHV1 ORF1 has been reported as a polypeptide of an unknown function, while ORF2 presents a polypeptide containing a UDP-glucose/sterol glucosyltransferase domain, a putative domain of permuted papain fold peptidases, an RdRP, and an RNA helicase domain. A defective RNA of 9816 nt was also observed and added to the characterisation of the genomic dynamics of ThHV1. This RNA presents the full ORF2 and its respective domains, but ORF1 is reduced in size [116].

In *Entomophthora*, only viruses in the families *Iflaviridae* (class *Pisoniviricetes*, order *Picornavirales*) and *Mitoviridae* (class *Howeltoviricetes*, order *Cryppavirales*) have received attention. The presence of viruses from the *Iflaviridae* family are arguably the most fascinating discovery from the transcriptomic datasets of *E. muscae* [78,79,117]. Iflaviruses are encapsidated monosegmented viruses with (+)ssRNA genomes ranging from *ca*. 8.8 to 10.1 kb in length that encompass two long overlapping ORFs that encode structural (CPs) and non-structural (helicase, protease, and RdRP) proteins. The *Iflaviridiae* are known to mostly infect insects, suggesting that in *Entomophthora*, the virus has shifted from insects during co-infections inside a dipteran host [78,79]. The names of these iflaviruses have not yet been established by the ICTV community, and since the organism these iflaviruses replicate in is not yet known, the name “*Entomophthovirus*” or names according to *E. muscae* and its original insect host (e.g., *E. muscae*–*M. domestica* iflavirus 1) have been suggested. Although studies point at these iflaviruses being able to survive in in vitro liquid fungal cultures, further research is required to show whether they are novel mycoviruses from the *Iflaviridae* or opportunistically transmitting to insects via phoresis with *E. muscae* conidia. The *E. muscae* iflaviruses show high-sequence homology with the Twyford virus, a *D. melanogaster*-associated iflavirus that was originally described from wild-caught *D. melanogaster* [118], which has since been shown to also have been infected with *E. muscae* [78,79]. To date, only a single *E. muscae* isolate has been reported without an *Iflavirus*, with some isolates harbouring two different species [78,79], suggesting the iflaviruses to be widely and obligately associated with *E. muscae*. Phylogenetic analyses showed that these *E. muscae* iflaviruses form a single clade, divergent from recognised insect-infecting *Iflaviridae* [78,79], but co-phylogenetic analyses did not support fungus–virus co-speciation [117]. The frequent co-infection of fungi and viruses has led to instances of transmission from the insect host to EPF [119]. Thus, the insect-acquired virus could evolve the ability to replicate in the new fungal host, as has been found in the deformed wing virus, an iflavirus infecting honey bees, which have phylogenetically diverged from the insect-infecting species and evolved the ability to infect and replicate in the fungal pathogen *Ascosphaera apis*, which causes honey bee chalkbrood disease [120]. Given the protoplast growth of *E. muscae*, the transition from being an insect-infecting virus to a fungal-associated virus could further be facilitated in *E. muscae*. However, further studies into other *Entomophthora* species would be critical to understand this cross-kingdom evolutionary history of fungal–viral symbiosis.

The *Mitoviridae* family comprises capsidless non-segmented viruses with (+)ssRNA genomes ranging from *ca*. 2 to 5 kb in length that encompass a single long ORF that encodes an RdRP. Mitoviruses lack true virions and replicate in the mitochondria of host fungi and plants. *E. muscae* was the first and most phylogenetically basal fungus found to harbour mitoviruses [28]. Indeed, every *E. muscae* isolate has been found to harbour one to eight mitoviruses, forming phylogenetically distinct clades. These mitoviruses have been named Entomophthora muscae mitovirus 1 through 8 (EnmuMV1–EnmuMV8). All *E. muscae* isolates are from the *Musca*-infecting (house fly) lineage harbour seven mitoviruses. Interestingly, an *E. muscae* isolate from the *Drosophila*-infecting lineage shared all seven mitoviruses, but also harboured an eighth mitovirus, as opposed to the *E. muscae* isolate from the *Delia*-infecting lineage (cabbage fly), which only had one mitovirus (EnmuMV3), suggesting *Entomophthora* lineage-specific mitovirus associations [28].

For the genus *Metarhizium*, no ssRNA viruses have been described to date.

## 4. Effects of Mycoviruses on the Fungal Host

Historically, the investigation of genomes, genes, proteins, and metabolites in entomopathogenic fungi has primarily focused on elucidating their pathogenic interactions with arthropods [121]. This interest stems from early observations of fungal spores on insect cadavers, referred to as white and green muscardine diseases, caused by *Beauveria* and *Metarhizium*, respectively, and the potential use of EPF. Several studies have focused on the search for strains with broader phenotypic characteristics in terms of their spectrum of action (e.g., at different temperatures, humidity, pH, and other abiotic factors) [122,123,124,125,126]. Others, with advances in molecular biology, have enabled precise manipulation of cellular processes to enhance the efficacy of these fungi as biological control agents. Approaches such as heterologous expression of proteins, gene editing, silencing, and overexpression have been employed to modify key molecular traits, especially those associated with virulence, metabolic efficiency, and stress tolerance [127,128,129,130].

The pioneer studies on the discovery of mycoviruses in phytopathogenic fungi and their impacts on host biology prompted the exploration of these viruses in EPF [131]. In view of the possibility that mycoviruses may actively participate in phenotypic changes in their fungal hosts, these studies aim to elucidate the biological roles of mycoviruses and their potential influence on fungal fitness, pathogenicity, and host interactions [132].

The description of the biological effects caused by the presence of mycoviruses in their hosts initially involves an essential step: obtaining isogenic strains. Early studies used strategies based on the biology of the fungi studied and the probability of obtaining ‘virus-free’ strains through conidiogenesis and isolation of single-spore cultures [34]. Obtaining isogenic strains can be observed spontaneously. After successive passages in laboratory culture media, studies observed an effect called sectorisation in strains of *M. anisopliae*, where the presence of viruses was altered in relation to the original colony [23,24]. Moreover, forced transmission techniques, such as heterokaryosis and anastomosis, also offered the possibility for obtaining an infected strain. For this, a virus-free strain is used as a recipient and, after interaction with a donor strain, spores are selected and analysed for the presence of viral genetic material. Successful transmission attempts were achieved by Martins et al. (1999) between strains of *M. flavoviride* from two different continents [133]. Finally, the use of molecules aiming at inhibiting protein synthesis and RNA replication has gained prominence in mycovirology, due to their high cure rates in different fungal groups. Drugs such as cycloheximide, a protein synthesis inhibitor that targets the translocation step translation, and ribavirin, a base analogue of either adenine or guanine, inducing mutations in RNA-dependent replication in RNA viruses, have been widely used [134]. Once potentially virus-free colonies are obtained, confirmation of curing is necessary. Classical methods such as the precipitation of dsRNA with lithium chloride or elution from CF-11 columns have been widely used in the past and are still useful for initial screening. However, nowadays, specific primers are used to amplify conserved regions, such as those present in RdRP, for confirmation by RT-PCR, bringing greater reliability, since the observation of bands on agarose gels can be biassed due to the inefficient extraction process or even by the detection limit of the technique itself. The main obstacle is that many mycoviral infections are latent and therefore do not have a specific or obvious phenotype, making it difficult to observe potential isogenic strains and increasing labour time.

**Figure 5:**
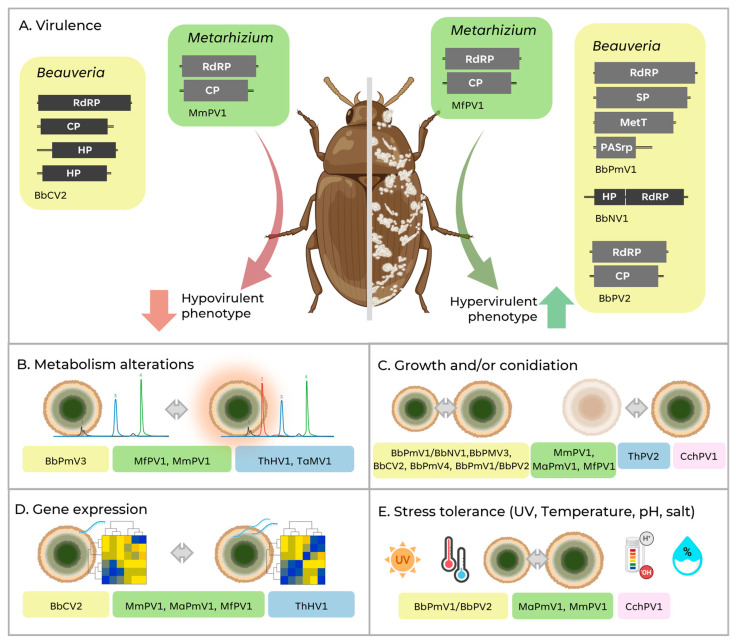
Overview of the biological impacts exerted by mycoviruses on entomopathogenic fungi. Colour coding refers to the fungal host genus: *Beauveria* (yellow), *Cordyceps* (lilac), *Metarhizium* (green), and *Trichoderma* (blue). Panel A illustrates the modulation of virulence by mycoviruses described in *Beauveria* and *Metarhizium*, with representative genome diagrams shown within colour-coded boxes according to their fungal host. Panels B–E summarise additional phenotypic effects (e.g., on sporulation, growth, thermotolerance, etc.), where virus acronyms appear in host-coloured boxes indicating the EPF genus in which the effect was observed. The full names of all viruses referred to by acronyms are listed in Supplementary Tables S2–S6. Created in BioRender. Camargo, M. (2025) https://BioRender.com/b0x8y5v (accessed on 2 December 2025).

Among the most widely investigated phenotypic factors regarding the presence or absence of mycoviruses in EPF, virulence, sporulation, and mycelial growth under different conditions are highlighted, due to their close relationship with EPF application as commercial products. Moreover, although hypervirulence is the desired factor through the transfer or expression of a virus, or part of it, in EPF, the hypovirulence phenotype may also indicate the possibility of increasing virulence by curing the viral elements responsible for this phenomenon. The next subheadings elucidate the biological effects triggered by mycoviruses in EPF and main phenotypes are summarised in Figure 5.

### 4.1. Beauveria spp. and Cordyceps spp. Biological Effects

The first evidence that mycoviruses could alter the *Beauveria* phenotype was reported in 2006 by Dalzoto et al. The authors showed that dsRNA elements in *B. bassiana* were directly responsible for hypovirulence, significantly reducing the fungal host’s ability of infecting insects. Isogenic fungal lines cured of the dsRNA elements demonstrated significant enhanced virulence against the brown stink bug (*Euschistus heros*) compared to its virus-infected counterparts [35]. Subsequent field surveys in China have linked mycoviruses to a hypovirulent phenotype of wild *B. bassiana* strains [135]. For instance, Beauveria bassiana chrysovirus 2 (BbCV2) was found as a prevalent “core” virus in many field isolates. BbCV2-infected strains showed reduced virulence against larvae of the corn borer (*Ostrinia furnacalis*), as compared to their virus-free counterparts [135]. Transcriptomic analysis of hypovirulent strains revealed a mechanistic basis, with a lowered expression of genes encoding cuticle-degrading enzymes and toxins, consistent with the observed drop in insect pathogenicity [135]. Conversely, some mycoviruses were found to enhance virulence on *Beauveria*. Kotta-Loizou and Coutts (2017) first reported a case of mild hypervirulence in *B. bassiana* in the Spanish EABb 92/11-Dm isolate harbouring two novel dsRNA viruses Beauveria bassiana polymycovirus 1 (BbPmV1) and Beauveria bassiana non-segmented virus (BbNV1), showing slightly faster growth and causing higher mortality in *Galleria mellonella* greater wax moth larvae as compared to a virus-cured isogenic line [37]. This initial discovery has since been followed by more dramatic examples of mycovirus-induced hypervirulence. A recent study by Rueda-Maíllo et al. (2025) showed that the Spanish *B. bassiana* strain EABb 01/126-Su, naturally coinfected with BbPmV1 and BbPV2, became significantly more virulent against *G. mellonella* larvae than a virus-free derivative [136]. Another report from China noted that BbPmV4, while reducing the fungus spore production, increased its virulence against *O. furnacalis* larvae [137].

In many hypervirulent cases, virus presence correlates with increased sporulation. For example, polymycovirus infection can dramatically elevate conidia yields: an unpublished study observed virus-infected *B. bassiana* isolates producing up to 96% more conidia than their virus-free isogenic lines [107]. Filippou et al. (2021) reported that two different polymycoviruses (BbPmV1 and BbPmV3) in *B. bassiana* strains EABb 92/11-Dm and ATHUM 4946 both led to significantly enhanced conidiation on standard Czapek-Dox agar (with sucrose/nitrate) [138]. This enhancement was reproducible across rich and minimal media, indicating that virus infection often boosts the fungus capacity to form conidia under a range of nutrient conditions. Moreover, the sporulation shift can depend on the intake of nutrients. Filippou et al., in 2021, also noted that when the carbon or nitrogen source was altered, the virus-mediated effect could be diminished or even reversed [138]. Conversely, some mycoviruses can suppress sporulation. As an example, *B. bassiana* infected with BbPmV4 exhibits noticeably lower conidial yields relative to virus-free controls [137].

*Beauveria* isolates harbouring polymycoviruses often display significantly increased radial growth on agar media. In the work of Filippou et al. (2021) [138], the colony diameter and biomass of virus-infected lines outpaced that of virus-free isogenic lines, sometimes substantially so, under standard culture conditions. Similarly, Kotta-Loizou and Coutts (2017) observed a mild growth advantage in a *B. bassiana* isolate containing dsRNA viruses, relative to its cured counterpart [37]. These findings align with a general pattern that certain mycoviruses (notably *Polymycoviridae* and some *Chrysoviridae*) elicit increased fungal growth in their hosts. At minimum, removing deleterious viruses can restore more robust growth. For example, Dalzoto et al. (2006) reported that curing *B. bassiana* of dsRNA not only restored insect pathogenicity but also the overall growth capacity of the fungus, suggesting that the virus had imposed some growth burden [35].

Rueda-Maíllo et al. (2025) provided striking evidence where the hypervirulent *B. bassiana* strain EABb 01/126-Su, carrying BbPmV1 and BbPV2, exhibited broad-spectrum stress resistance as compared to virus-free lines [136]. The mycovirus-containing strain could germinate and grow across an expanded temperature range and showed increased tolerance to osmotic stress, water stress (desiccation), and ultraviolet (UV)-B radiation. Interestingly, the same virus-infected strain also displayed enhanced antagonistic ability against *T. harzianum* [136]. In addition to fungal antagonism, some mycoviruses appear to shift the ecological role of *Beauveria* strains toward improved plant interactions. Zhang et al. (2024) demonstrated that *B. bassiana* infected with BbCV2, a hypovirulence-associated chrysovirus, exhibited significantly enhanced antagonism against phytopathogens including *Botrytis cinerea* and *Sclerotinia sclerotiorum* [139]. Moreover, the virus-infected strains showed improved root colonisation and plant growth promotion traits, alongside an upregulated expression of genes associated with antimicrobial secondary metabolites [139]. These findings suggest that certain mycoviruses can attenuate insect virulence while simultaneously increasing the strain’s potential utility as a biocontrol agent against plant pathogens.

In *Cordyceps*, recent studies have begun to elucidate the effects of partitiviruses on their fungal hosts. For instance, CchPV1, a member of the *Gammapartitivirus* genus, was found to alter fungal development and stress tolerance in *Cordyceps chanhua*. CchPV1 infection was shown to slow the host’s growth rate while increasing conidiation and the formation of fruiting bodies. However, the infection also weakened the host’s multi-stress tolerance, indicating that partitiviruses can have complex and multifaceted effects on their *Cordyceps* hosts [96].

### 4.2. Metarhizium spp. Biological Effects

In *M. anisopliae* V291, an isogenic strain obtained through successive subcultures on solid medium (V291C), no difference in virulence against the aphid *Myzus persicae* was observed when compared with its virus-infected counterpart (V291B). Both clones displayed similar pathogenicity levels, with aphid mortalities of 95.2 ± 16.9% and 88.2 ± 3.4%, respectively, despite the presence of virus-like particles and dsRNA segments being detected only in V291B [23]. Conversely, alterations in colony morphology and increased conidial production were reported in a virus-free spontaneous mutant strain (RJd), compared to the parent strain (RJc) of *M. anisopliae* [24]. For the same species, a virus-free strain (STA2-1c2) was obtained through monosporic cultures, which, compared to the virus-infected strain, showed a slight increase in colony size and spore production on potato dextrose agar (PDA) medium amended with dodine and notably, a significant reduction in virulence against crickets (*Gryllus domesticus*) [34].

In *M. acridum*, several treatments were attempted in an effort to cure *M. acridum* CG291. However, single-conidium subculture, elevated incubation temperature, and cycloheximide treatment were all unsuccessful in virus eradication. Hyphal anastomosis between the auxotrophic mutants CG291-arg and CG442-met was successfully conducted. Analysis comparing the original CG291 strain and its infected version showed no difference in virulence against the grasshopper *Rhammatocerus schistocercoides* [133]. Subsequent studies conducted on the same isogenic strains found no significant difference in the activity of the pathogenesis-related-protein 1 (PR-1L), a protein with enzymatic activity of importance for the fungal infection cycle. However, changes were observed in conidia production, which was greater for CG442-met (−dsRNA) than for CG442-met7 and CG442-met8 (+dsRNA) [50].

Studies aimed at eliminating dsRNA viruses from *Metarhizium* spp. strains revealed that cycloheximide was ineffective in eradicating dsRNA segments. By contrast, hyphal tip subculturing resulted in the loss of some dsRNA segments in *M. anisopliae* ESALQ 26. Complete elimination of dsRNA was achieved in strains ESALQ 866, ESALQ 1256, and ESALQ 1595 through either monoconidial or hyphal tip subculturing. Fungal variants free from or harbouring dsRNA exhibited comparable tolerance to heat and UV stress, indicating limited evidence to suggest that these mycoviruses have a negative impact on the biological traits of their fungal host [59].

In *M. majus*, horizontal transmission of Metarhizium majus partitivirus 1 (MmPV1) was achieved, following the co-culture of the MmPV1-infected strain RCEF0578 with the MmPV1-free strain RCEF0577. Transmission occurred through mycelial fusion at the colony margin, followed by subculturing on a solid medium until sporulation and the isolation of single spores from the recipient strain. Inter-simple sequence repeat PCR (ISSR-PCR) was employed to distinguish the two strains prior to repeated co-cultivation. Isogenic MmPV1-infected single-spore isolates exhibited reduced conidiation, decreased tolerance to heat shock and UV-B irradiation, and downregulation of the genes associated with these traits. MmPV1 infection also impaired fungal virulence, affecting hydrophobicity, adhesion, and cuticular penetration. Secondary metabolite production was altered, with decreases in triterpenoids and metarhizins A and B and increases in nitrogen- and phosphorus-containing compounds. Notably, when individual MmPV1 proteins (RdRP and CP) were expressed, no alterations in phenotype were observed [140].

As in MmPV1, isogenic virus-infected lines exhibited significant alterations in biological traits as compared to their virus-free counterparts in Metarhizium anisopliae polymycovirus 1 (MaPmV1) [107]. Isogenic lines derived from single spore cultures were obtained by vortex-mixing conidial suspensions in Tween-80 and filtering through sterile non-woven fabric. In MaPmV1-infected strains, biological comparisons revealed enhanced growth rates and increased conidiation, supported by the upregulation of genes related to these traits. However, MaPmV1 also increased the host’s sensitivity to UV-B irradiation, correlating with the downregulation of DNA damage repair genes. Unlike MmPV1, MaPmV1 did not significantly affect *M. anisopliae* virulence. Despite these observations, overexpression of individual MaPmV1 proteins failed to induce any notable phenotypic changes [107].

Another successful example of viral transfection between fungal strains highlighted the potential of Metarhizium flavoviride partitivirus 1 (MfPV1) to enhance the fungal performance as a biocontrol agent. PEG-mediated protoplast transfection of commercial strains of *M. anisopliae* and *M. pingshaense* with MfPV1 significantly increased their virulence against two major lepidopteran pests, the diamondback moth (*Plutella xylostella*) and the fall armyworm (*Spodoptera frugiperda*). MfPV1-infected strains also exhibited enhanced conidial production and upregulated PR genes, including those encoding an adhesin-like protein, hydrolytic enzymes, and destruxin synthetase [141].

### 4.3. Trichoderma spp. Biological Effects

Since the genus *Trichoderma* has different characteristics from the previous genera, such as the ability to mycoparasitise, there are different approaches and findings regarding the presence or absence of mycoviruses in their fungal hosts. One of the main characteristics investigated, along with conidiation and growth in *Trichoderma*, is the interaction with other microorganisms, including species of economic and food importance, such as the basidiomycete *Pleurotus*, and phytopathogenic species including *Fusarium* and *Botrytis* spp. The approaches, although outside the scope of insect pest control, include an extra factor of interest for evaluation in phenotypes of *Beauveria* and *Metarhizium*, since both also participate in fungus–plant associations and can interact with invading phytopathogenic fungi.

An initial study on *Trichoderma* evaluated the presence of viruses in different strains and found patterns compatible with viruses from the *Hypoviridae* family in the *T*. *harzianum* M2 strain. Through selection of single-spore colonies, 2 out of 30 showed absence of the viral elements initially observed [142]. No changes were observed in development and conidiation patterns. In addition, the elimination of TaMV1 from a *T*. *atroviride* strain was also achieved through single-spore selection. As observed in the previous study, no effects on morphological parameters, such as pigmentation, sporulation, and growth rate, were observed in the virus-free/containing comparison [27].

In *T*. *harzianum*, transmission by anastomosis was used to transfer ThPV1, and the infected strains were compared to their virus-free counterparts. The inhibition level against the oyster mushroom, *P. ostreatus*, and the phytopathogen *Rhizoctonia solani* was increased when filtrates were prepared from *T*. *harzianum* cultures infected with ThPV1. More profoundly, a significant increase in the activity of the antifungal enzyme of β-1,3-glucanase was observed in the ThPV1-infected strain, an important enzymatic factor for the mycoparasitic activity of *T*. *harzianum* [94]. Conversely, the alphapartitivirus TaPV1 had no significant impact on virus-free strains of *T*. *atroviride* obtained by single-spore selection followed by hyphal tipping [98].

Another study of isogenic strains obtained by viral transmission revealed the biological role of ThHV1 and ThHV1S (a defective RNA of ThHV1). Firstly, the specific role of ThHV1-S was observed, as it negatively interfered with the mycelial growth of strain T70-D, as well as decreasing the inhibitory potential against the phytopathogen *Botrytis cinerea*. These phenotypes were also observed in the *T. harzianum* isolates T-68 and *T*. *koningiopsis* isolate T-51 transfected with ThHV1 and/or ThHV1S [116]. Subsequent analyses indicated patterns of transcriptomics and metabolomics related to the effect previously observed using filtrates from infected strain T-51-13, indicating mechanisms behind the phenotype of the increased inhibition of phytopathogenic fungal infection [143].

Virus elimination in *Trichoderma* was also achieved using ribavirin and protoplasting/regeneration. Slight differences in mycelial growth were observed when comparing *T*. *harzianum* strain 525 and its respective counterpart 525-F on specific media. Moreover, the absence of TaMV1 was implied by decreased biomass production by the host and lower antagonistic activity against the pathogen *F*. *oxysporum* in planta [85]. Conversely, the infection caused by TaPV2 was related to the slightly increased but significant capacity of *T*. *harzianum* to inhibit growth of *F*. *oxysporum* [144].

An important result was observed in isogenic *T*. *atroviride* strains obtained by selecting single-spore colonies. The impact observed for the presence of TaMV1-NFCF377 (related to the *Fusagraviridae* family) is associated with enhanced antifungal efficacy against the phytopathogen *R. solani*, but not for the edible mushroom *P. ostreatus*. Despite this, no immediate effects such as growth or sporulation have been reported [90].

### 4.4. Entomophthora spp. Biological Effects

Although a high diversity and prevalence of viruses have been identified in the *Entomophthora* [76,77], the understanding of the biological effects they have on the fungus or the insect host is almost entirely lacking. To date, only insights into the symbiosis with the divergent *Iflaviridae* species have been reported [78,79,117]. Iflavirus reads are found in *E. muscae* from RNA sequenced in both in vivo and in vitro cultures, showing that the virus is capable of survival in liquid fungal in vitro cultures [78,79]. In infected *Drosophila melanogaster*, an increase in reads mapping to iflaviral genomes in late stages of infection have been observed [78].

Because *E. muscae* consistently harbours iflaviruses and also behaviourally manipulates infected fly hosts, the idea that the virus could be involved in behaviourally manipulating the host insects is not excluded [78,79]. For example, DNA viruses in the *Baculoviridae* family are known to induce comparable summiting phenotypes before death in lepidopteran larvae [145]. Furthermore, an *Iflaviridae* species has previously been shown to induce behavioural change in an insect [146], showing that these genomically simple iflaviruses could be manipulating the behaviour of *Entomophthora*-infected insects. To identify the role of *E. muscae* iflaviruses, one main hurdle presents itself: finding a virus-free isolate in wild-caught flies or obtaining a virus-free isolate in in vitro cultures. As *E. muscae* is cultured in liquid media where it does not sporulate, the only feasible option available to obtaining a virus-free culture is to isolate single conidium from sporulating cadavers, which can then be cultured in liquid medium. Given the usually slow and temperamental growth in vitro (2–6 weeks with millions of conidia), obtaining a virus-free culture will likely be long and tedious, but essential to uncovering the interaction of the near-ubiquitous *E. muscae* iflaviral symbiont.

Overall, considering the viral groups with the highest number of reported phenotypic descriptions in EPF, partitiviruses and polymycoviruses display distinct levels and patterns of impact on their fungal hosts (Tables S2–S6). Partitiviruses, on one hand, show no clear trend, as they can either increase or reduce virulence, growth, and sporulation, depending on the specific virus–host combination. These effects suggest a broader modulation of host metabolism and resource allocation, variably influencing developmental processes, stress tolerance, and pathogenicity. Polymycoviruses, on the other hand, tend to produce more consistent outcomes, frequently associated with enhanced virulence, mycelial growth, and conidiation, although are sometimes accompanied by reduced stress tolerance or sporulation. Collectively, these patterns indicate that both groups modulate host physiology in distinct ways. Partitiviruses seem to induce variable, context-dependent effects that can either favour or impair host performance, whereas polymycoviruses are more frequently linked to enhanced growth and virulence, suggesting a tendency to boost the overall fitness of entomopathogenic fungi under certain conditions. Nevertheless, we emphasise that the number of described viruses, even within these families, remains limited. Broader analyses involving these and other viral taxa are still required to establish a clearer correlation between the viral family and the phenotypic effects observed in their fungal hosts.

## 5. Knowledge Gaps and Future Perspectives in EPF Mycovirology

### 5.1. Molecular Advances on Mycovirology

Mycoviruses are seminal as tools for inducing phenotypic changes in their fungal hosts. Since their discovery, significant advances have been made, from the observation of their effects during the golden age of mycovirology to the successful biocontrol of *C. parasitica* in chestnut trees [2].

Promising advances in the description of new viruses are also based on the development of new sequencing techniques. In addition to NGS platforms with greater genomic coverage and sequencing depth, innovations aimed at sequencing and describing dsRNA sequences have attracted much attention. Fragmented primer-linked double-stranded RNA sequencing (FLDS) is a technology that facilitates the detection of mycovirus genomes in a homology-independent manner [147]. The method, described by Urayama et al. (2016), makes it possible to sequence viral genetic material in a circular form, with no ends and therefore no need for subsequent sequence descriptions, such as RNA ligase-mediated rapid amplification of cDNA ends (RLM-RACE) [147]. The reduction in the number of steps makes it possible to discover new viral taxa more quickly.

Other bioinformatics tools have also enabled the in-depth discovery of unconventional segments of known viruses. Previous techniques are being seen as limited because the scope of the databases is insufficient, given the commonly used parameters. By using strict parameters, sequences with little similarity to those already deposited may not be recognised as viral material. In this sense, by using more comprehensive parameters and smaller sequences, such as those that are well conserved and present in untranslated regions (UTRs), viral sequences can be captured from large databases as genome-mining, even if the ORFs present in them do not have much similarity to those that are already deposited. Tools such as iUTR have recently been applied to the yet-unrecognised genus “Jivivirus” and have revealed up to seven new segments for a viroid hitherto considered to be tri-segmented [148,149].

Moreover, aiming at the use of mycovirus sequences as molecular tools and the clarification of molecular basis behind phenotypic alterations, research is increasingly focused on identifying the specific viral and fungal components responsible for phenotypical alterations observed in the viruses’ hosts. In *Aspergillus*, a widely described fungus with a diverse incidence of mycoviruses and their respective biological effects, the RNA silencing mechanism proved to be important for the process of cause and effect in phenotypic alterations. In this context, mycoviruses can be both triggers and targets, as well as suppressors of RNA silencing. As for the defence mechanisms of mycoviruses against host silencing systems, the processes of protection of viral genetic material, inhibition of Dicer and Argonaute proteins, or high turnover of vsiRNAs are reported. Effects relating to the process of virus-RNAi system interaction are observed mainly at the level of transcriptomic alteration, as reviewed by Battersby et al. (2024) [14].

Finally, experiments expressing individual viral proteins or combinations of proteins are helping to elucidate the molecular mechanisms of virus–host interactions. Techniques such as heterologous expression, using basic plasmid systems and cloning by *Agrobacterium tumefaciens*, make it possible to express single viral proteins and understand the specific roles of each virus sequence in their hosts. In *M. majus*, although effects were observed when the virus was transferred in its entirety, no significant changes were seen after expression of the individual MmPV1 proteins [140].

### 5.2. Environmental Implications on Mycoviruses Transmission

Despite the advancements in mycovirus genomic descriptions, there are still significant gaps in the understanding of the mechanisms behind viral transmission and their implications for the evolution and distribution of viral families in fungal taxa.

**Figure 6:**
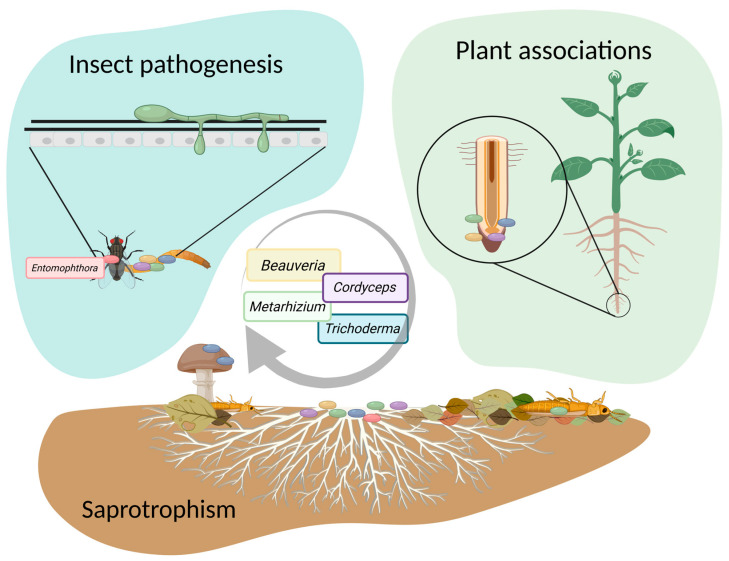
Lifecycle of *Beauveria*, *Cordyceps*, *Entomophthora*, *Metarhizium*, and *Trichoderma*, highlighting potential sources and pathways for mycovirus infections. The diagram illustrates the various stages in the lifecycle of EPF where mycoviruses could potentially be transmitted or acquired, underscoring the ecological interactions that facilitate virus infection within different environmental and host contexts. Created in BioRender. Camargo, M. (2025) https://BioRender.com/e5dnahg (accessed on 2 December 2025).

The known existence of viruses in different components of the environments in which EPF are found (Figure 6) raises even more questions about the potential for viral transfer in these environments. *Beauveria* (along with the *Cordyceps* complex), *Metarhizium*, and *Trichoderma* have different life cycles: acting as saprophytic fungi—degrading organic compounds in the soil; endophytic—participating in mycorrhizal interactions or endosymbionts in plants; and entomopathogenic—capable of suppressing the defence barrier of arthropods and using insects as a way of dispersing and controlling populations [150,151,152].

Viral transfer is an essential mechanism for virus dispersal and has been used for biological control strategies in the past [2,153]. In laboratory research, as well as enabling the development of isogenic strains by horizontal transmission, viral transfer can also explain how these viruses are distributed naturally. Previous studies have reported the similarity of viral nucleotide and amino acid sequences of more than 90% in *Sclerotinia homoeocarpa* and *Ophiostoma novo*-*ulmi* [154]. Other studies have also revealed the ability of viruses described in *Entoleuca* (Entoleuca hypovirus 1—EnHV1) to infect a strain of *Rosellinia necatrix* [155] and, for the Botrytis porri RNA virus 1, the co-occurrence in *S*. *sclerotiorum* [156].

In *Trichoderma*, the presence of viruses capable of replicating in another fungal genus has already been observed [82], revealing a scarcely explored potential for mycovirus dispersal beyond species-specific levels. Furthermore, extrapolating to the environmental context of fungi, recent studies have demonstrated the influence of microenvironments on the induction of viral transfer. By recreating conditions such as a high concentration of proline, it was possible to observe in vitro processes that were previously observed in planta. Viral transfer was even increased to strains considered incompatible, changing the perspective regarding the possibility of natural dispersal of mycoviruses [157]. Finally, studies have shown that the inter-kingdom relationships, including mycoviruses, can cross over the fungus–fungus interaction. Nerva et al. (2017) reported the replicative capacity in plant cells of two virus species belonging to the *Partitiviridae* and the former *Totiviridae* families, with no indication of adaptation to the host or alteration of nucleotide sequences [158]. In addition, a DNA virus identified in the phytopathogenic fungus *S*. *sclerotiorum* was reported as being capable of infecting a mycophagous insect when the larvae fed on virus-infected fungi. Notably, viruliferous adults could transmit the mycovirus by transovarial transmission and potentially play a part as mycovirus vectors [159]. This finding points to the ecological potential of viral transmission between fungal species and provides a basis for the development of new biocontrol strategies, such as the dissemination of hypervirulence-inducing viruses in populations of EPF, mirroring the success observed in *C. parasitica*.

Regarding the viral classification in terms of cross-kingdom distribution, despite the *Ghabrivirales* order, which mainly comprises mycoviruses-obligatory dsRNA viruses, the *Mononegavirales*, *Reovirales*, and *Martellivirales* orders, which comprise (+) or (−) ssRNA viruses, have a wide dispersion, including fungi, animals, and plants [153].

Moreover, the fungal–insect interactions should not be examined solely from an EPF perspective. Within the tripartite plant–insect–fungi system, research has demonstrated that fungi manipulate insect behaviour through volatile organic compounds (VOCs) to enhance phytopathogenic fungal dispersal [160,161]. Indeed, mycoviruses can suppress fungal secondary metabolite biosynthesis, consequently altering insect attraction to mycovirus-infected fungi targeting insect-born dissemination [161]. The wide occurrence of mycovirus in several pathosystems add an additional level of complexity to the multitrophic interactions. These observations highlight how mycovirus and chemical ecology studies can illuminate novel biocontrol strategies for complex field interactions.

As previously discussed, ThMV1 exhibits dual effects in *T. harzianum*—enhancing plant growth promotion while reducing its biocontrol efficacy. This suggests viral-mediated modulation of fungal secondary metabolites involved in plant growth regulation. Such tripartite symbioses (the hot springs panic grass (*Dichanthelium lanuginosum*)-*Curvularia protuberata*-Curvularia thermal tolerance virus (CThTV)) have also been implicated in thermal tolerance [162]. These mutualistic interactions align with the ‘Russian doll’ model proposed by Lerer and Shlezinger (2022), wherein mycoviruses fine-tune host virulence and ecological adaptability [132]. In this context, the mycovirus-induced phenotypic alterations in fungi position host–virus interactions as a crucial research area in modern virology, where these interactions offer promising applications as novel biocontrol agents and bioinputs to sustainable agriculture, bioindustrial tools for fermentation and bioprocessing and alternative therapeutic strategies to address antifungal resistance crises.

Finally, a major experimental challenge lies in validating the practical application of virus-mediated phenotypic changes. Data on the dissemination of mycoviruses intentionally introduced into fungal strains prepared in the laboratory in environmental or agricultural conditions remains scarce, leaving crucial questions unanswered about the stability and scalability of producing mycovirus-infected EPF strains for large-scale spore application in crop protection. The central challenge now is to turn mycoviruses into tools, rather than just subjects of study, in the EPF mycovirology scenario.

## Supplementary Materials

The following supporting information can be downloaded at: https://www.mdpi.com/article/10.3390/v17121593/s1. Table S1: Incidence of mycoviruses in entomopathogenic fungi. Table S2: Mycoviruses characterised in *Beauveria* and their properties. Table S3: Mycoviruses characterised in *Cordyceps* and their properties. Table S4: Mycoviruses characterised in *Entomophthora* and their properties. Table S5: Mycoviruses characterised in *Metarhizium* and their properties. Table S6: Mycoviruses characterised in *Trichoderma* and their properties. Refs. [163,164,165,166,167,168,169,170,171,172,173] are cited in Supplementary Materials.
