# Supplementary material for: Mycoviruses in Entomopathogenic Fungi
Source: Viruses. 2025 Dec 8;17(12):1593. doi: 10.3390/v17121593 (PMC12737583; doi:10.3390/v17121593)
Supplement: Supplementary file 1 [file viruses-17-01593-s001.zip › viruses-3897564-supplementary.pdf]

*Review*

# Mycoviruses in Entomopathogenic Fungi

**Matheus da Silva Camargo [1,2], Sam Edwards [3], Maressa O. Henrique [4], Urja Solanki [5], Tae Young Shin [6], Bo Huang [7], Henrik H. De Fine Licht [8], Marcio C. Silva-Filho [4], Augusto Schrank [1], Robert H. A. Coutts [5] and Ioly Kotta-Loizou [2,5,*]**

[1] Centro de Biotecnologia, Programa de Pós-graduação em Biologia Celular e Molecular, Universidade Federal do Rio Grande do Sul, Porto Alegre 91501-970, RS, Brazil; silva.camargo@ufrgs.br (M.d.S.C.); aschrank@ufrgs.br (A.S.)
[2] Department of Life Sciences, Imperial College London, London SW7 2AZ, UK
[3] Laboratory of Virology, Wageningen University & Research, 6708 PB Wageningen, The Netherlands; sam.edwards@wur.nl
[4] Departamento de Genética, Escola Superior de Agricultura Luiz de Queiros, Universidade de São Paulo, Piracicaba 13418, SP, Brazil; maressahenrique@usp.br (M.O.H.); mdcsilva@usp.br (M.C.S.-F.)
[5] Department of Clinical, Pharmaceutical & Biological Science, School of Health, Medicine and Life Sciences, University of Hertfordshire, Hatfield AL10 2AB, UK; u.solanki@herts.ac.uk (U.S.); r.coutts@herts.ac.uk (R.H.A.C.)
[6] Department of Agricultural Biology, College of Agricultural and Life Sciences, Jeonbuk National University, Jeonju 54896, Republic of Korea; tyshin@jbnu.ac.kr
[7] Anhui Provincial Key Laboratory of Biological Control, Anhui Agricultural University, Hefei 230036, China; bhuang@ahau.edu.cn
[8] Section for Organismal Biology, Department of Plant and environmental Sciences, University of Copenhagen, 1871 Frederiksberg, Denmark; hhdefinelicht@plen.ku.dk
* Correspondence: i.kotta-loizou2@herts.ac.uk or i.kotta-loizou13@imperial.ac.uk

## Supplementary Materials

**Table S1.** Incidence of mycoviruses in entomopathogenic fungi.

| Species | No. of isolates analysed | No. of isolates infected | Location | Reference |
|---|---|---|---|---|
| *Beauveria amorpha* | 1 | 0 | Spain | [38] |
| *Beauveria bassiana* | 12 | 2 | Canada | [34] |
| | 13 | 2 | Brazil (culture collections) | [35] |
| | 73 | 40 | Spain and Portugal | [25] |
| | 8 | 6 | New Zealand | [36] |
| | 75 | 16 | Brazil, Ecuador, Greece, Portugal, Russia, Spain, Syria, Uzbekistan | [37] |
| | 43 | 12 | Spain, France, and Denmark | [38] |
| | 73 | 8 | Korea | [41] |
| | 28 | 8 | China | [40] |

| | | | | |
|---|---|---|---|---|
| *Beauveria brongniartii* | 7 | 0 | Korea | [41] |
| *Beauveria caledonica* | 1 | 1 | New Zealand | [36] |
| *Beauveria pseudobassiana* | 5 | 0 | Spain | [38] |
| | 2 | 0 | Korea | [41] |
| *Beauveria varroae* | 3 | 0 | Spain | [38] |
| *Entomophthora muscae* [a] | 5 | 5 | RNA-seq public databases[b] | [28] |
| | 5 | 5 | Denmark and Germany | [117] |
| | 1 | 1 | Denmark | [79] |
| | 1 | 1 | USA | [78] |
| *Metarhizium anisopliae* | 41 | 2[c] | World[c] | [23] |
| | 7 | 3 | Brazil | [22] |
| | 3 | 2 | Oman and Trinidad | [34] |
| | 73 | 28 | Canada | [34] |
| | 5 | 4 | Brazil | [58] |
| | 35 | 22 | Brazil (ESALQ collection) | [59] |
| | 7 | 0 | Spain, Italy and Georgia | [38] |
| *Metarhizium flavoviride* | 6 | 5 | Australia, Benin, Chad, Mali | [34] |
| | 7 | 5 | Brazil, Tanzania, Nigeria and Australia | [133] |
| | 1 | 0 | Denmark | [38] |
| *Metarhizium robertsii* | 1 | 0 | Brazil (ESALQ collection) | [59] |
| | 5 | 0 | Spain and Denmark | [38] |
| *Metarhizium brunneum* | 4 | 0 | Spain and Denmark | [38] |
| *Metarhizium guizhouense* | 2 | 0 | Spain | [38] |
| *Metarhizium majus* | 1 | 0 | Denmark | [38] |
| *Metarhizium pemphigi* | 7 | 2 | Korea | [41] |
| *Metarhizium pinghaense* | 2 | 2 | Korea | [41] |
| *Metarhizium rileyi* | 1 | 1 | Korea | [41] |
| *Trichoderma spp.* | 156 | 2 | China | [163] |
| | 307 | 32 | Korea | [142] |
| | 163 | 4[d] | China and Mongolia | [108] |
| *Trichoderma harzianum* | 39 | 19 | Italy | [164] |

| | | | | |
|---|---|---|---|---|
| *Trichoderma gamsii* | 29 | 7 | | |
| *Trichoderma hamatum* | 9 | 6 | | |
| *Trichoderma tomentosum* | 5 | 2 | | |
| *Trichoderma samuelsii* | 4 | 1 | | |
| *Trichoderma spirale* | 12 | 1 | | |
| *Trichoderma velutinum* | 5 | 0 | | |
| *Trichoderma virens* | 4 | 0 | | |
| *Trichoderma koningii* | 2 | 0 | | |
| *Trichoderma koningiopsis* | 2 | 0 | | |
| *Trichoderma asperellum* | 1 | 0 | | |
| *Cordyceps* sp. | 1 | 1 | Brazil | [49] |
| *Cordyceps amoene-rosea* | 2 | 1 | Brazil | |
| *Cordyceps farinosa* | 1 | 1 | United States | |
| *Cordyceps fumosorosea* | 6 | 3 | Brazil, USA, France, and Nepal | |
| | 12 | 3 | Brazil, USA, Mexico and India | [165] |
| | 2 | 1 | Korea | [41] |

a – For *Entomophthora muscae*, only RNA-seq and public transcriptome data are available; no population-based isolate studies have yet been reported.
b – Genomes assembled from public databases.
c – From Finland and Brazil.
d – *T. barbatum* and *T. harzianum* species.

**Table S2. Mycoviruses characterised in *Beauveria* and their properties.** The mycovirus name, the *Beauveria* spp. strain from which each virus was isolated, taxonomic classification (family and genus), and genome type are provided. For each viral RNA segment, the size in base pairs or nucleotides (bp or nt) with respective GenBank accession number, the encoded protein length in amino acids (aa), as well as its putative function, are listed. Reported effects of the mycovirus on the fungal host phenotype are summarised, alongside the detection methods used.

| Mycovirus | Strain/Isolate | Taxonomy | Genome type | Segment size (bp or nt) | ORF product (aa) | Sequencing method | Effect on host phenotype | Reference |
|---|---|---|---|---|---|---|---|---|
| Beauveria bassiana RNA virus 1 (BbRV1) | EABb 06/02-Su | *Totiviridae* | dsRNA 1 segment | 5,228; HE572591 | CP (742); CCC42234<br>RdRP (834); CCC42235 | Sanger | N.D. | [25] |
| Beauveria bassiana victorivirus 1 (BbVV1) | ICMP#6887 | *Totiviridae Victorivirus* | dsRNA 1 segment | 5,327; NC_024151 | CP (759); YP_009032632<br>RdRP (843); YP_009032633 | Sanger | N.D. | [36] |
| Beauveria bassiana non-segmented virus 1 (BbNV1) | EABb 92/11-Dm | "Unirnavirus" | dsRNA 1 segment | 3,218; LN610699 | HP (315); CEF90231<br>RdRP (585); CEF90232 | Sanger | ↑ Virulence<br>↑ Mycelial growth | [37,83] |
| Beauveria bassiana partitivirus 1 (BbPV1) | IMI 331273 | *Partitiviridae Gammapartitivirus* | dsRNA 2 segments | 1,771; LN896303<br>1,601; LN896304 | RdRP (539); CUS18591<br>CP (440); CUS18592 | Sanger | N.D. | [37] |
| Beauveria bassiana partitivirus 2 (BbPV2) | IMI 392612 | *Partitiviridae Gammapartitivirus* | dsRNA 2 segments | 1,801; LN896305<br>1,548; LN896304 | RdRP (539); CUS18593<br>CP (432); CUS18594 | Sanger | ↑ Virulence<br>↑ UV-B resistance<br>↑ Drought tolerance<br>↑ Temperature tolerance<br>↑ Antagonistic activity | [37,136] |
| Beauveria bassiana small Narna-like virus (BbSNLV) | SP R 159 | *Narnaviridae Narnavirus* | ssRNA 1 segment | 1,689; LT627647 | RdRP (509); SCW25778 | Sanger | N.D. | [37] |

| | | | | | | | | |
|---|---|---|---|---|---|---|---|---|
| Beauveria bassiana polymycovirus 1 (BbPmV1) | EABb 92/11-Dm | *Polymycoviridae*<br>*Polymycovirus* | dsRNA<br>4 segments | 2,425; LN896307<br>2,260; LN896308<br>1,921; LN896309<br>1,373; LN896310 | RdRP (775); CUS18595<br>HP (704); CUS18596<br>MetT (610); CUS18597<br>PASrp (306); CUS18598 | Sanger | ↑ Virulence<br>↑ Mycelial growth<br>↑ UV-B resistance<br>↑ Drought tolerance<br>↑ Temperature tolerance<br>↑ Antagonistic activity | [37,136] |
| Beauveria bassiana chrysovirus 1 (BbCV1) | GXsk1011 | *Chrysoviridae*<br>*Alphachrysovirus* | dsRNA<br>4 segments | 3,478; MK279433<br>3,143; MK279434<br>3,069; MK279435<br>2,770; MK279436 | RdRP (1,115); AZT88571<br>CP (976); AZT88572<br>HP (964); AZT88573<br>HP (851); AZT88574 | RNA-seq | N.D. | [102] |
| Beauveria bassiana non-segmented RNA virus 1 (BbRNV1) | ARSEF 8028 | Unassignated | dsRNA<br>1 segment | 3,170; MK279499 | HP (315); AZT88648<br>RdRP (590); AZT88649 | RNA-seq | N.D. | [102] |
| Beauveria bassiana partitivirus 3 (BbPV3) | RCEF5853 | *Partitiviridae*<br>*Epsilonpartitivirus* | dsRNA<br>2 segments | 1,856; MN116721<br>1,719; MN116720 | RdRP (584); QFP40245<br>CP (500); QFP40246 | RNA-seq | N.D. | [166] |
| Beauveria bassiana polymycovirus 4 (BbPmV4) | BbOFJY | *Polymycoviridae*<br>*Polymycovirus* | dsRNA<br>6 segments | 2,427; MW385785<br>2,280; MW385786<br>2,015; MW385787<br>1,106; MW385788 | RdRP (768); QRF54813<br>Scaffold protein (699); QRF54814<br>MetT (617); QRF54815<br>PASrp (265); QRF54816 | RNA-seq | morphology<br>↓ sporulation<br>↑ virulence | [39,137,167] |

| | | | | | | | | |
|---|---|---|---|---|---|---|---|---|
| | | | | 867; MW385789 | Unknown (202); QRF54817 | | | |
| | | | | 794; MW385790 | PASrp (161); QRF54818 | | | |
| Beauveria bassiana chrysovirus 2 (BbCV2) | BbOFZK152 | *Chrysoviridae Betachrysovirus* | dsRNA 4 segments | 3,441; MW314841 | RdRP (1,114); QQZ01307 | RNA-seq | morphology gene expression ↑ biomass ↓ virulence ↑ inhibition against phytopathogenic fungi | [103,135,139] |
| | | | | 2,779; MW314842 | CP (805); QQZ01308 | | | |
| | | | | 2,925; MW314843 | HP (768); QQZ01309 | | | |
| | | | | 2,688; MW314844 | HP (777); QQZ01310 | | | |
| Beauveria bassiana bipartite mycovirus 1 (BbBV1) | RCEF1446 | Unassignated | dsRNA 2 segments | 2,026; MW265927 | RdRP (596); UDZ92910 | RNA-seq | N.D. | [168] |
| | | | | 1,810; MW265928 | CP (322); UDZ92911 | | | |
| Beauveria bassiana polymycovirus 3 (BbPmV3) | ATHUM 4946 | *Polymycoviridae Polymycovirus* | dsRNA 6 segments | 2,401; LR991938 | RdRP (767); CAD7829823 | Sanger | Metabolism changes ↑ growth (dependent specific media) | [138] |
| | | | | 2,240; LR991939 | Scaffold protein (697); CAD7829824 | | | |
| | | | | 1,989; LR991940 | MetT (615); CAD7829825 | | | |
| | | | | 1,131; LR991941 | PASrp (268); CAD7829826 | | | |
| | | | | 937; LR991942 | Unknown (170); CAD7829827 | | | |
| | | | | 8,65; LR991943 | Unknown (205); CAD7829828 | | | |
| Beauveria bassiana victorivirus 2 (BbVV2) | RCEF6864 | *Totiviridae Victorivirus* | dsRNA 1 segment | 5,233; OR126353 | CP (742); WMJ90927 | RNA-seq | N.D. | [40] |
| | | | | | RdRP (834); WMJ90928 | | | |

| | | | | | | | | |
|---|---|---|---|---|---|---|---|---|
| Beauveria bassiana bipartite virus 2 (BbBV2) | RECF6899 | Unassignated | dsRNA<br>2 segments | 2,164;<br>ON938188 | RdRP (664);<br>WEI52980 | RNA-seq | N.D. | [40] |
| | | | | 1,760;<br>ON938189 | CP (317);<br>WEI52981 | | | |
| Beauveria bassiana negative-strand RNA virus 1 (BbNSV1) | GusB2 | related to *Mymonaviridae* | ssRNA<br>1 segment | 6,169;<br>OR737625 | RdRP (1,949);<br>WPE03610 | RNA-seq | N.D. | [99] |
| Beauveria bassiana orthocurvulavirus 1 (BbOCV1) | GusB2 | related to *Curvulaviridae* | dsRNA<br>2 segments | 2,164;<br>OR737623 | RdRP (664);<br>WPE03609 | RNA-seq | N.D. | [99] |
| | | | | 1,765;<br>OR737624 | CP (317);<br>WPE03614 | | | |

N.D. = Not determined.

BbNV1 and BbPMV1 biological effects were determined in combination.

**Table S3. Mycoviruses characterised in *Cordyceps* and their properties.** The mycovirus name, the *Cordyceps* spp. strain from which each virus was isolated, taxonomic classification (family and genus), and genome type are provided. For each viral RNA segment, the size in base pairs or nucleotides (bp or nt) with respective GenBank accession number, the encoded protein length in amino acids (aa), as well as its putative function, are listed. Reported effects of the mycovirus on the fungal host phenotype are summarised, alongside the detection methods used.

| Mycovirus | Strain/Isolate | Taxonomy | Genome type | Segment Size (bp or nt) | ORF product (aa) | Sequencing method | Effect on host phenotype | Reference |
|---|---|---|---|---|---|---|---|---|
| Isaria javanica chrysovirus 1 (IjCV1)* | NB IFR-19 | *Chrysoviridae* | dsRNA 4 segments | 3,593; KX898416 | RdRP (1,117); APR73428 | RNA-seq | N.D. | [26] |
| | | | | 3,175; KX898417 | CP (974); APR73429 | | | |
| | | | | 3,165; KX898418 | Unknown (977); APR73430 | | | |
| | | | | 2,874; KX898419 | Put. Protease (843); APR73431 | | | |
| Cordyceps chanhua alternavirus 1 (CcAV1) | RCEF6000 | *Alternaviridae, Alternavirus* | dsRNA 3 segments | 3,512; OK481552 | RdRP (1127); UPH33984 | RNA-seq | N.D. | [104] |
| | | | | 2,655; OK481553 | HP1 (831); UPH33985 | | | |
| | | | | 2,415; OK481554 | HP2 (731); UPH33986 | | | |
| Cordyceps chanhua partitivirus 1 (CchPV1) | RCEF5997 | *Partitiviridae, Gammapartitivirus* | dsRNA 2 segments | 1,784; OP727721 | RdRP (540); WBW48344 | RNA-seq | ↓ Mycelial growth<br>↑ Conidiation<br>↑ Formation of fruiting bodies<br>↓ UV-B resistance<br>↓ Heat tolerance<br>↓ Salt tolerance<br>↓ Osmotic tolerance<br>↓ Antioxidant tolerance | [96] |
| | | | | 1,563; OP727722 | CP (435); WBW48345 | | | |

| | | | | | | | | |
|---|---|---|---|---|---|---|---|---|
| Cordyceps militaris partitivirus 1 (CmPV1) | RCEF7506 | *Partitiviridae, Betapartitivirus* | dsRNA 2 segments | 2,206; PP155648 | RdRP (670); WUV41264 | RNA-seq | N.D. | [95] |
| | | | | 2,256; PP155647 | CP (652); WUV41263 | | | |
| Cordyceps javanica negative-strand RNA virus 1 (CjNRSV1) | NB7 | *Phenuiviridae, Laulavirus* | dsRNA 3 segments | 7,252; PP544309 | RdRP (2,384); WYM67035 | RNA-seq | N.D. | [113] |
| | | | | 2,401; PP544310 | MP (692); WYM67036 | | | |
| | | | | 1,117; PP544311 | NC (254); WYM67037 | | | |

N.D. = Not determined

* Following recent reclassifications accommodating *Isaria* species to the *Cordyceps* genus (according to Kepler *et al*, 2017 [43]).

**Table S4. Mycoviruses characterised in *Entomophthora* and their properties.** The mycovirus name, the *Entomophthora* spp. strain from which each virus was isolated, taxonomic classification (family and genus), and genome type are provided. For each viral RNA segment, the size in base pairs or nucleotides (bp or nt) with respective GenBank accession number, the encoded protein length in amino acids (aa), as well as its putative function, are listed. Reported effects of the mycovirus on the fungal host phenotype are summarised, alongside the detection methods used.

| Mycovirus | Strain/Isolate | Taxonomy | Genome type | Segment Size (bp or nt) | ORF product (aa) | Sequencing method | Effect on host phenotype | Reference |
|---|---|---|---|---|---|---|---|---|
| Entomophthora muscae mitovirus 1 (EnmuMV1)* | KVL-14-117 | *Mitoviridae; Mitovirus* | (+)ssRNA 1 segments | 2,804; MK682513.1 | RdRP (757); QCF24445.1 | RNA-seq | N.D. | [28] |
| Entomophthora muscae-Musca domestica iflavirus 1 (EmmdIV1)* | KVL21-40 | *Iflaviridae; Iflavirus* | (+)ssRNA 1 segment | 8,706; PQ468742 | Polyprotein (2,901); XKQ11312.1 | RNA-seq | N.D. | [117] |
| Entomophthora partitivirus D** | Won | *Partitiviridae; Partitivirus* | dsRNA 1 segment | 2,106; MK231054.1 | RdRP (555); QED42916.1 | RNA-seq | N.D. | [77] |
| Entomophthora benyvirus E** | Cho | *Benyviridae; Benyvirus* | (+)ssRNA | 6,325; MK231113.1 | Put. RdRP (2,062); QED42962.1 | RNA-seq | N.D. | [77] |
| Entomophthora rhabdovirus A** | Won | *Rhabdoviridae; Rhabdovirus* | (-)ssRNA 1 segment | 6,934; MK231099.1 | Put. RdRP (2,249); QED42948.1 | RNA-seq | N.D. | [77] |
| Entomophthora narnavirus A** | Won | *Narnaviridae; Narnavirus* | (+)ssRNA 1 segment | 2,838; MK940812.1 | RdRP (914); QED43053.1 | RNA-seq | N.D. | [77] |
| Entomophthora virgavirus A** | Won | *Virgaviridae; Virgavirus* | (+)ssRNA 1 segment | 10,376; MK231110.1 | Polyprotein (2,186); QED42956.1<br>HP (650); QED42957.1<br>HP (195); QED42958.1<br>HP (190); QED42959.1 | RNA-seq | N.D. | [77] |

N.D. = Not determined; Put. RdRP: Putative RdRP; *Complete CDS.; **Partial genome.

**Table S5. Mycoviruses characterised in *Metarhizium* and their properties.** The mycovirus name, the *Metarhizium* spp. strain from which each virus was isolated, taxonomic classification (family and genus), and genome type are provided. For each viral RNA segment, the size in base pairs or nucleotides (bp or nt) with respective GenBank accession number, the encoded protein length in amino acids (aa), as well as its putative function, are listed. Reported effects of the mycovirus on the fungal host phenotype are summarised, alongside the detection methods used.

| Mycovirus | Strain/Isolate | Taxonomy | Genome type | Segment Size (bp or nt) | ORF product (aa) | Sequencing method | Effect on host phenotype | Reference |
|---|---|---|---|---|---|---|---|---|
| Metarhizium brunneum partitivirus 1 (MbPV1) | RCEF0736 | *Partitiviridae*, "Epsilonpartitivirus" | dsRNA 2 segments | 1,829; MN395473 | RdRP (573); QHB49873 | RNA-seq | N.D. | [29] |
| | | | | 1,720; MN395474 | CP (496); QHB49874 | | | |
| Metarhizium brunneum partitivirus 2 (MbPV2) | RCEF0736 | *Partitiviridae, Gammapartitivirus* | dsRNA 2 segments | 1,775; MT584790 | RdRP (539); QTC11257 | RNA-seq | N.D. | [97] |
| | | | | 4,568; MT584791 | CP (430); QTC11258 | | | |
| Metarhizium brunneum polymycovirus 1 (MbPmV1) | RCEF0736 | *Polymycoviridae; Polymycovirus* | dsRNA 4 segments | 2,421; OP524132 | RdRP (767); WBL45227 | RNA-seq | N.D. | [106] |
| | | | | 2,280; OP524133 | Unknown (699); WBL45228 | | | |
| | | | | 2,018; OP524134 | MetT (615); WBL45229 | | | |
| | | | | 1,153; OP524135 | PASrp (180); WBL45230 | | | |
| Metarhizium brunneum bipartite mycovirus 1 (MbBV1) | RCEF0766 | Unassigned | dsRNA 2 segments | 1,987; ON350778 | RdRP (597); UPO93687 | RNA-seq | N.D. | [169] |
| | | | | 1,642; ON350779 | CP (308); UPO93688 | | | |
| Metarhizium anisopliae | M5 | *Totiviridae, Victorivirus* | dsRNA 1 segment | 5,353; OP959068 | CP (757); WCS16032 | RNA-seq | N.D. | [81] |

| | | | | | | | | |
|---|---|---|---|---|---|---|---|---|
| victorivirus 1 (MaVV1) | | | | | RdRP(839); WCS16033 | | | |
| Metarhizium majus partitivirus 1 (MmPV1) | RCEF0578 | *Partitiviridae, Gammapartitivirus* | dsRNA 2 segments | 1,721; OL518956<br>1,407; OL518957 | RdRP (538); UPO93685<br>CP (415); UPO93686 | RNA-seq | ↓ Conidiation<br>↓ Heat tolerance<br>↓ UV-B tolerance<br>↓ Virulence<br>Changes in secondary metabolism | [140] |
| Metarhizium anisopliae polymycovirus 1 (MaPmV1) | RCEF3284 | *Polymycoviridae, Polymycovirus* | dsRNA 4 segments | 2,421; OP627094<br>2,259; OP627095<br>1,917; OP627096<br>1,395; OP627097 | RdRP (775); WGN91658<br>HP (704); WGN91659<br>MetT (610); WGN91660<br>PASrp (305); WGN91661 | RNA-seq | ↑ Mycelial growth<br>↑ Conidiation<br>↑ Expression of growth and conidiation-related genes<br>↓ UV-B tolerance | [107] |
| Metarhizium flavoviride partitivirus 1 (MfPV1) | Mf_KF18 | *Partitiviridae, Gammapartitivirus* | dsRNA 2 segments | 1,775; MH143600<br>1,575; MH143601 | RdRP (538); AZU96334<br>CP (440); AZU96335 | RNA-seq | ↑ Virulence<br>↑ Conidiation<br>↑ expression of virulence genes<br>Changes in secondary metabolism | [141] |
| Metarhizium robertsii polymycovirus 1 (MrPmV1) | SDSET-20.42 | *Polymycoviridae, Polymycovirus* | dsRNA 4 segments | 2,342; PV166302<br>2,231; PV166303<br>1,885; PV166304<br>1,341; PV166305 | RdRP (758); XQD59753<br>Scaffold protein (716); XQD59754<br>MetT (610); XQD59755<br>PASrp (322); XQD59756 | RNA-seq | N.D. | [170] |
| Metarhizium acridum unirnavirus 1 (MaUV1) | CG423 | *Amalgaviridae, Unirnavirus* | dsRNA 1 segment | 2,907; PV797947.1 | HP (317); XXJ30516.1<br>RdRP (609); XXJ30517.1 | RNA-seq | N.D. | [84] |

N.D. = Not determined.

**Table S6. Mycoviruses characterized in *Trichoderma* and their properties.** The mycovirus name, the *Trichoderma* spp. strain from which each virus was isolated, taxonomic classification (family and genus), and genome type are provided. For each viral RNA segment, the size in base pairs or nucleotides (bp or nt) with respective GenBank accession number, the encoded protein length in amino acids (aa), as well as its putative function, are listed. Reported effects of the mycovirus on the fungal host phenotype are summarised, alongside the detection methods used.

| Mycovirus | Strain/Isolate | Taxonomy | Genome type | Segment Size (bp or nt) | ORF product (aa) | Sequencing method | Effect on host phenotype | Reference |
|---|---|---|---|---|---|---|---|---|
| Trichoderma atroviride mycovirus 1 (TaMV1) | NFCF028 | *Fusagraviridae** | dsRNA; 1 segment | KX821737; 8,566 | Put. Struct./gag (1,429); APU87539<br>RdRP (1,214); APU87540 | Sanger | No changes | [27] |
| Trichoderma harzianum partitivirus 1 (ThPV1) | NFCF319 | *Partitiviridae, Betapartitivirus* | dsRNA 2 segments | 2,289; MG973751<br>2,245; MG973752 | RdRP (722); AZL30773<br>CP (659); AZL30774 | RNA-seq | ↑ Enzyme activity<br>↑ Antagonistic activity | [94] |
| Trichoderma atroviride partitivirus 1 (TaPV1) | NFCF394 | *Partitiviridae, Alphapartitivirus* | dsRNA 2 segments | 2,023; MH921573<br>2,012; MH921574 | RdRP (614); AYQ58321<br>CP (577); AYQ58322 | RNA-seq | No changes | [98] |
| Trichoderma asperellum dsRNA Virus 1 (TaRV1) | JLM45-3 | *Fusagraviridae** | dsRNA; 1 segment | 9,838; MG897472 | HP (1,502); AXR76116<br>RdRP (1,327); AXR76117 | RNA-seq | N.D. | [171] |
| Trichoderma koningiopsis totivirus 1 (TkTV1) | Mg10 | *Totiviridae, Totivirus* | dsRNA; 1 segment | 4,712; MK993478 | CP (687); QGA70770<br>RdRP (747); QGA70771 | RNA-seq | N.D. | [82] |
| Trichoderma harzianum hypovirus 1 (ThHV1) | T-70* | *Hypoviridae, Betahypovirus* | (+)ssRNA 2 segments | 11,214; MN172262 | Polyprotein (443); QGA30968<br>Polyprotein (2,793); QGA30969 | Sanger | ↓ Mycelial growth<br>↓ Conidiation<br>↓ Mycoparasitic activity<br>Changes in secondary metabolism | [116] |

| | | | | | | | | |
|---|---|---|---|---|---|---|---|---|
| | | | | 9,816b; MN172263 | Polyprotein (2,807); QGA30970 | | | |
| Trichoderma harzianum mycovirus 1 (ThMV1) | HB40525 | Unclassified | dsRNA; 1 segment | 3,160; MH155602 | RdRP (582); AYU71187<br>CP (379); AYU71188 | RNA-seq | ↑ Antagonistic activity<br>↓ Plant growth induction<br>↑ Biomass production | [85] |
| Trichoderma harzianum bipartite mycovirus 1 (ThBMV1) | 137 | Unclassified | dsRNA 2 segments | 2,088; MH536648<br>1,634; MH536649 | RdRP (631); AXU24203<br>HP (314); AXU24204 | RNA-seq | N.D. | [172] |
| Trichoderma atroviride mycovirus 1 (TaMV1) | NFCF377 | *Fusagraviridae** | dsRNA; 1 segment | 9,584; MT450869 | Struct./gag (1,429); QMU26426<br>RdRP (1,236); QMU26427 | RNA-seq | ↑ Antagonistic activity<br>Changes in secondary metabolism | [90] |
| Trichoderma harzianum Partitivirus (ThPV2) | T673 | *Partitiviridae* | dsRNA 2 segments | 1,693; OL457022<br>1,458; OL457023 | RdRP (524); UVB68789<br>CP (375); UVB68790 | RNA-seq | ↑ Antagonistic activity<br>↑ Conidiation and chlamydospore formation<br>↓ Plant growth induction | [144] |
| Trichoderma harzianum hypovirus 2 (ThHV2) | M6 | *Hypoviridae*, *Alphahypovirus* | (+)ssRNA 1 segment | 13,813; MW863664 | Polyprotein (4,118); UDP60414 | RNA-seq | N.D. | [173] |
| Trichoderma barbatum polymycovirus 1 (TbPMV1) | HB40111 | *Polymycoviridae*, *Polymycovirus* | dsRNA 4 segments | 2,420; OM307406<br>2,127; OM307407<br>1,909; OM307408 | RdRP (766); WAA18813<br>HP (678); WAA18814<br>MetT (613); WAA18815 | RNA-seq | N.D. | [108] |

| | | | | | | | | |
|---|---|---|---|---|---|---|---|---|
| | | | | 1,041; OM307409 | HP (260); WAA18816 | | | |

N.D. = Not determined

*Further studies were carried in *T. koningiopsis* T-51 strain and alterations in secondary metabolites production were observed [143].
